# A tissue-specific role for intraflagellar transport genes during craniofacial development

**DOI:** 10.1371/journal.pone.0174206

**Published:** 2017-03-27

**Authors:** Elizabeth N. Schock, Jaime N. Struve, Ching-Fang Chang, Trevor J. Williams, John Snedeker, Aria C. Attia, Rolf W. Stottmann, Samantha A. Brugmann

**Affiliations:** 1 Division of Plastic Surgery, Department of Surgery, Cincinnati Children’s Hospital Medical Center, Cincinnati, Ohio, United States of America; 2 Division of Developmental Biology, Department of Pediatrics, Cincinnati Children’s Hospital Medical Center, Cincinnati, Ohio, United States of America; 3 Department of Craniofacial Biology, University of Colorado School of Dental Medicine, Aurora, Colorado, United States of America; 4 Division of Human Genetics, Department of Pediatrics, Cincinnati Children’s Hospital Medical Center, Cincinnati, Ohio, United States of America; Justus Liebig Universitat Giessen, GERMANY

## Abstract

Primary cilia are nearly ubiquitous, cellular projections that function to transduce molecular signals during development. Loss of functional primary cilia has a particularly profound effect on the developing craniofacial complex, causing several anomalies including craniosynostosis, micrognathia, midfacial dysplasia, cleft lip/palate and oral/dental defects. Development of the craniofacial complex is an intricate process that requires interactions between several different tissues including neural crest cells, neuroectoderm and surface ectoderm. To understand the tissue-specific requirements for primary cilia during craniofacial development we conditionally deleted three separate intraflagellar transport genes, *Kif3a*, *Ift88* and *Ttc21b* with three distinct drivers, *Wnt1-Cre*, *Crect* and *AP2-Cre* which drive recombination in neural crest, surface ectoderm alone, and neural crest, surface ectoderm and neuroectoderm, respectively. We found that tissue-specific conditional loss of ciliary genes with different functions produces profoundly different facial phenotypes. Furthermore, analysis of basic cellular behaviors in these mutants suggests that loss of primary cilia in a distinct tissue has unique effects on development of adjacent tissues. Together, these data suggest specific spatiotemporal roles for intraflagellar transport genes and the primary cilium during craniofacial development.

## Introduction

Primary cilia are ubiquitous, microtubule-based extensions that protrude off a plethora of cell types throughout development. Interest in primary cilia biology has grown exponentially over the last decade, mostly due to the identification of ciliopathies, a growing class of human syndromes that occur as a result of aberrant cilia function [1]. Although there is no established phenotypic criterion for diagnosis of a ciliopathy, it has been hypothesized that a ciliopathy could be defined on the basis of common phenotypic presentations. The initial suggestion for ciliopathic presentation consisted of a combination of nine common phenotypes including: retinitis pigmentosa, renal cystic disease, polydactyly, situs inversus, mental retardation, hypoplasia of the corpus callosum, Dandy-Walker malformation, posterior encephalocele and hepatic disease [1]. These common phenotypic characteristics suggest that certain tissues are particularly sensitive to the loss of primary cilia.

More recently, the craniofacial complex has also been identified as an organ system particularly sensitive to the loss of primary cilia [2–11]. Approximately 30% of all ciliopathies are primarily defined by their craniofacial phenotype, which frequently includes cleft lip/palate, craniosynostosis, micrognathia/aglossia and midfacial hyperplasia [5]. The craniofacial complex is comprised of tissues from various embryonic origins including the neuroectoderm, neural crest and surface ectoderm. Defects in any one of these tissues can lead to severe craniofacial disorders, and null mutations would be expected to be most severe, as they would represent the combinatorial loss of function in all tissues.

One of the barriers to gaining a clearer understanding of the etiology of ciliopathic phenotypes has been conflicting reports regarding phenotypes and molecular readouts of ciliopathic mutants. Specifically, there have been contradictory reports regarding how loss of cilia affects Hedgehog (Hh) signaling [12–16]. Some studies reported that loss of functional cilia produced a loss-of-Hh target gene expression [14, 16], while other data showed that loss of functional cilia produced a gain-of-Hh target gene expression [12, 13]. Conflicting data also exists when examining the role of individual ciliary proteins in Hh signaling [6, 15]. The basis for these conflicts are still under investigation; however, it is clear that the molecular mechanism rendering the cilia non-functional (e.g., which ciliary protein is lost or mutated) and the tissue in which this occurs has a major influence on the eventual effect on Hh target gene expression. This hypothesis is supported by the wide spectrum of phenotypes observed in ciliopathic animal models [5].

The axoneme of the cilium extends from the basal body and protrudes off the apical surface of the cell. Several ciliopathies are caused by the loss of axonemal extension, which requires intraflagellar transport (IFT) proteins. IFT proteins are divided into two classes: anterograde proteins (Class B) which carry molecular cargo from cell body to the ciliary tip (e.g., KIF3A and IFT88), and retrograde proteins (Class A) which carry molecular cargo from the ciliary tip back to the cell body (e.g., TTC21B). Our previous work examined how the loss of KIF3A in cranial neural crest cells impacted craniofacial development [6, 17]. We identified several craniofacial phenotypes in *Kif3a*^*f/f*^*;Wnt1-Cre* mutants including bifid nasal septum, cleft lip/palate, micrognathia, aglossia and dental defects. This cadre of craniofacial phenotypes indicated that KIF3A-dependent ciliogenesis played a neural crest-specific role during craniofacial development and differentiation. To follow up on these findings we decided to ask two main questions. First, would other tissues that contribute to the craniofacial complex be affected by loss of *Kif3a* or primary cilia? Second, would loss of other intraflagellar transport genes in these tissues recapitulate the *Kif3a* phenotype?

## Results

### Tissue specific Cre-drivers allow for conditional knockout of ciliary genes in the tissues that contribute to the craniofacial complex

**Fig 1:**
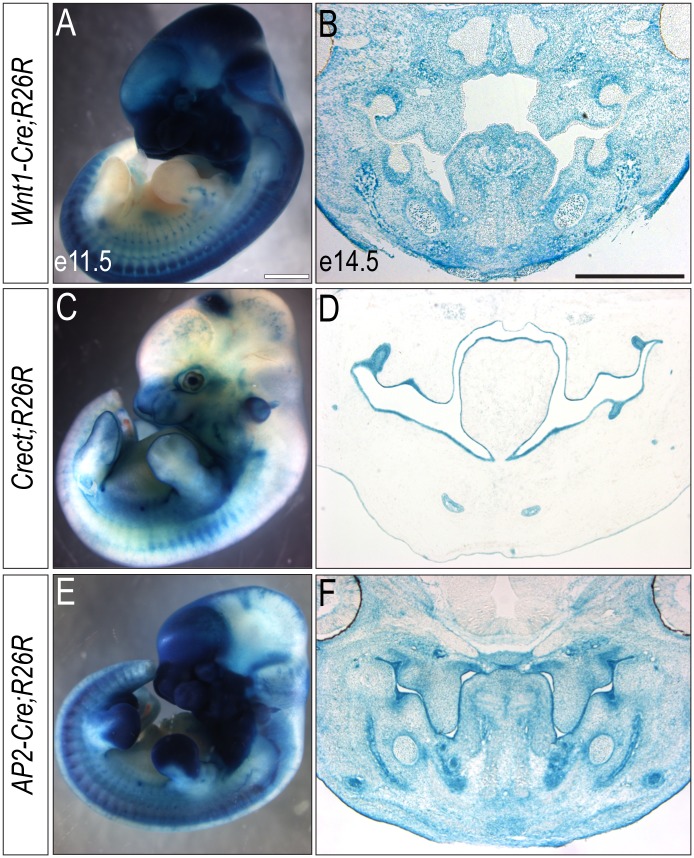
Various Cre-drivers used to knockout ciliary genes within the tissues of the craniofacial complex. (A, C, E) e11.5 whole-mount and (B, D, F) e14.5 sectioned (A, B) *Wnt1-Cre; R26R*, (C, D) *Crect;R26R*, or (E, F) *AP2-Cre;R26R* embryos stained for β-gal. Scale bars: 1.5 mm (A, C, E) and 750 μm (B, D, F).

Development of the craniofacial complex requires reciprocal interactions between many tissues for proper development [18]. To examine how cilia function in individual tissues or a combination of tissues within the craniofacial complex, we utilized three different Cre drivers to conditionally ablate ciliary genes. First, we employed the *Wnt1-Cre* driver, which recombines within the dorsal neural tube giving rise to neural crest cells (NCCs) and a portion of dorsal neuroectoderm in the developing midbrain (Fig 1A and 1B)[19–21]. Neural crest cells make up the majority of the cranial mesenchyme and make numerous contributions to the craniofacial complex, most notably to the facial mesenchyme and skeletal elements. Second, we utilized the *Crect* driver [22] to target recombination to cells within the surface ectoderm (Fig 1C and 1D). The developing surface ectoderm houses many signaling centers that are important for directing craniofacial development such as the frontonasal ectodermal zone (FEZ) and the nasal pits [23–27]. The *Crect* driver recombines in the surface ectoderm of the developing face (n = 17; 53%); however, we also observed a less defined recombination pattern (n = 15; 47%; S1A,B). Finally, we implemented the *AP2-Cre* driver [28]. *AP2-Cre*-mediated recombination occurs in NCCs, surface ectoderm and neuroectoderm (Fig 1E and 1F). The neuroectoderm, particularly the forebrain, serves as the scaffold upon which the face develops [29]. In addition to physically supporting facial development, the neuroectoderm also serves as an important signaling center, supplying the face with essential molecular inputs that help to guide midfacial development [23, 30]. Temporal onset and spatial domains of recombination for all three drivers used are summarized in Table 1. To confirm the efficacy and specificity of all three drivers we carried out immunostaining at both e11.5 and e14.5 for the ciliary marker Arl13b on *Kif3a*^*f/f*^;*Wnt1-Cre*, *Kif3a*^*f/f*^;*Crect* and *Kif3a*^*f/f*^;*AP2-Cre* embryos and found cilia were absent from neural crest alone, surface ectoderm alone and both neural crest and surface ectoderm, respectively (S2 Fig). To address the role of individual ciliary proteins and the cilia in each of these tissues, we next conditionally ablated three distinct IFT ciliary genes (*Kif3a*, *Ift88* and *Ttc21b*) and analyzed the resulting facial phenotype.

**Table 1:** Summary of temporal onset and spatial area of recombination within the craniofacial complex.

Cre	Cre-activity initiation	Tissue where recombination is initiated	Craniofacial tissues with recombination at e11.5	Craniofacial tissues with recombination at e14.5	References
*Wnt1-Cre*	~e8.0 (3 somites)	Anterior neural plate: NCCs and midbrain	NCC derived mesenchyme in developing mandible, palatal shelves and frontonasal prominence	Nasal septum cartilage, Meckel’s cartilage, NCC derived mesenchyme in palatal shelf, tooth bud, mandible and tongue	Chai *et al*., 2000; Jacques-Fricke *et al*.,2012
*Crect*	~e8.5 (10 somites)	Ectoderm of first pharyngeal arch and frontonasal prominence	Oral ectoderm, surface ectoderm, nasal pit ectoderm	Oral ectoderm, tooth bud epithelium, surface ectoderm, nasal epithelium	Tavares *et al*., 2012; Forni *et al*., 2011;Reid *et al*., 2011
*AP2-Cre*	~e7.0	Anterior neural folds (5 somites), pharyngeal arch ectoderm (by 10 somites), neural crest cells (5–10 somites)	NCC derived mesenchyme in developing mandible, palatal shelves and frontonasal prominence, oral ectoderm, surface ectoderm, nasal pit ectoderm	Nasal septum cartilage, Meckel’s cartilage, NCC derived mesenchyme in palatal shelf, tooth bud, mandible and tongue. Oral ectoderm, surface ectoderm, nasal epithelium	Macatee *et al*., 2003; Mašek *et al*., 2016

### Loss of *Kif3a* in tissue-specific domains of the craniofacial complex generates a range of phenotypes

**Fig 2:**
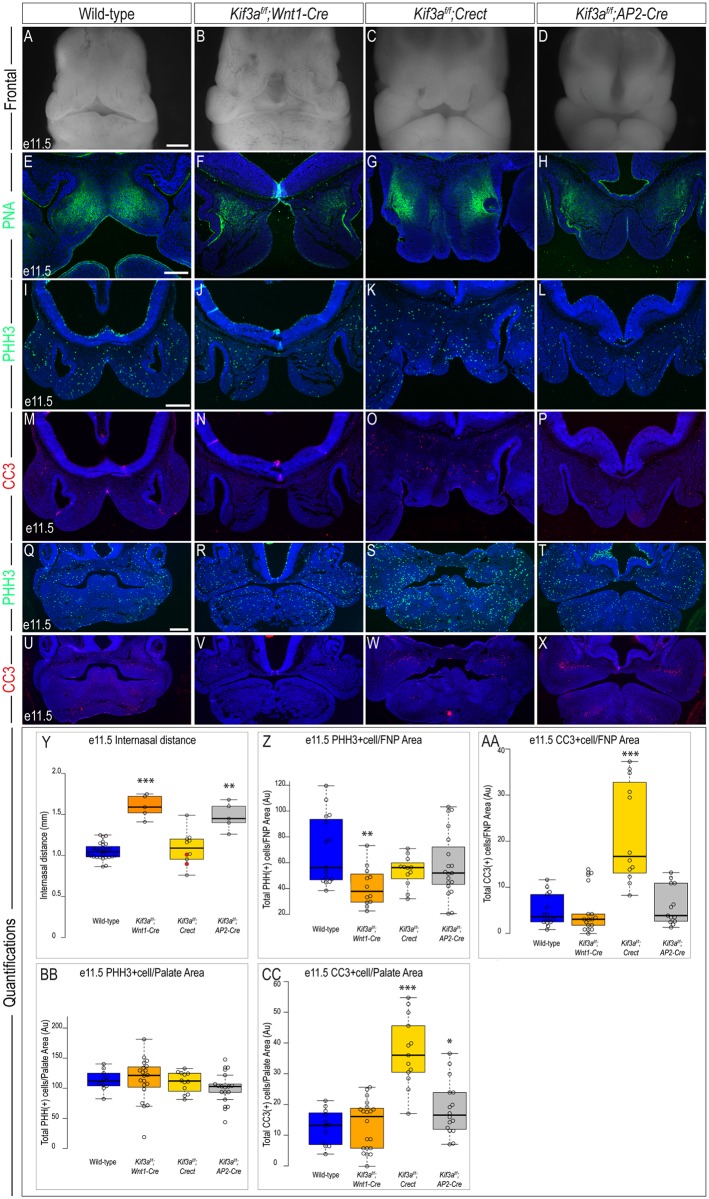
Analysis of e11.5 *Kif3a* conditional mutants. Frontal view of e11.5 (A) wild-type, (B) *Kif3a*^*f/f*^*;Wnt1-Cre*, (C) *Kif3a*^*f/f*^*;Crect*, and (D) *Kif3a*^*f/f*^*;AP2-Cre* embryos. PNA staining in e11.5 sections of (E) wild-type, (F) *Kif3a*^*f/f*^*;Wnt1-Cre*, (G) *Kif3a*^*f/f*^*;Crect*, (H) *Kif3a*^*f/f*^*;AP2-Cre* embryos. PHH3 staining in e11.5 frontonasal sections of (I) wild-type (n = 15), (J) *Kif3a*^*f/f*^*;Wnt1-Cre* (n = 12), (K) *Kif3a*^*f/f*^*;Crect* (n = 12), (L) *Kif3a*^*f/f*^*;AP2-Cre* (n = 19) embryos. CC3 staining in e11.5 frontonasal sections of (M) wild-type (n = 14), (N) *Kif3a*^*f/f*^*;Wnt1-Cre* (n = 17); (O) *Kif3a*^*f/f*^*;Crect* (n = 12), (P) *Kif3a*^*f/f*^*;AP2-Cre* (n = 13) embryos. PHH3 staining in e11.5 palatal sections of (Q) wild-type (n = 10), (R) *Kif3a*^*f/f*^*;Wnt1-Cre* (n = 20), (S) *Kif3a*^*f/f*^*;Crect* (n = 12), (T) *Kif3a*^*f/f*^*;AP2-Cre* (n = 17) embryos. CC3 staining in e11.5 palatal sections of (U) wild-type (n = 12), (V) *Kif3a*^*f/f*^*;Wnt1-Cre* (n = 20); (W) *Kif3a*^*f/f*^*;Crect* (n = 13), (X) *Kif3a*^*f/f*^*;AP2-Cre* (n = 16) embryos. (Y-CC) Quantification of measured values represented as box plots where the median and interquartile range are displayed. Red dots on the graph represent *Kif3a*^*f/f*^*;Crect* mutants that did not display a craniofacial phenotype and were not included in the rest of the analyses (2/10). For each genotype, *n = 3* embryos were utilized for PHH3/CC3 analyses. P-values: (*) 0.05≥P>0.01; (**) 0.01≥P>0.001; (***) P≤0.001. Scale bars: (A-D) 500 μm, (E-H) 200 μm, (I-P) 225 μm, and (Q-X) 350μm.

KIF3A is a member of the kinesin superfamily and functions as an anterograde IFT protein [31]. To examine the role of *Kif3a* in tissues contributing to the craniofacial complex we conditionally excised *Kif3a* using each of the three drivers detailed above and examined craniofacial domains frequently affected in ciliopathies. Our previous work identified a widened midline, as determined by an increased distance between the nasal pits (internasal distance), as the distinguishing feature of *Kif3a*^*f/f*^;*Wnt1-Cre* embryos [6, 17]. As expected, the first obvious phenotype in *Kif3a*^*f/f*^;*Wnt1-Cre* embryos at e11.5 was a significant increase in the internasal distance (n = 5), relative to wild-type embryos (n = 28) (Fig 2A, 2B and 2Y). In contrast, *Kif3a*^*f/f*^;*Crect* embryos, in which *Kif3a* was lost in the surface ectoderm, did not have a significant difference in internasal distance when compared to wild-type embryos (Fig 2C and 2Y, n = 10). In addition to the *Crect* recombination pattern reported in Fig 1, we also observed *Kif3a*^*f/f*^;*Crect* mutants with the alternate, broader pattern of recombination (S1C and S1D Fig). Regardless of which recombination pattern was present, the craniofacial phenotypes generated were relatively similar (S1C–S1F Fig). In *Kif3a*^*f/f*^;*AP2-Cre* embryos, in which *Kif3a* was lost in NCCs, surface ectoderm and some neuroectoderm, a significant increase in the internasal distance and medially rotated nasal pits were observed (Fig 2D and 2Y).

We continued our analysis of each mutant at e11.5 to determine if loss of *Kif3a* in different tissues and tissue combinations had an effect on cell differentiation, cell proliferation or cell death. We first examined the earliest stages of cell differentiation and formation of the skeletal condensations by performing peanut agglutinin (PNA) immunostaining. We observed domains of PNA staining in *Kif3a*^*f/f*^*;Wnt1-Cre* mutants that were laterally shifted, relative to wild-type embryos (n = 3) (Fig 2E and 2F). Similar to the pattern observed in *Kif3a*^*f/f*^*;Wnt1-Cre* mutants, a lateral displacement of the early condensations was observed in *Kif3a*^*f/f*^*;Crect* (n = 3) and *Kif3a*^*f/f*^*;AP2-Cre* (n = 3) mutants (Fig 2G and 2H). Thus, despite a shifted domain, the process of differentiation did not appear to be impaired in any of the ciliary mutants.

To determine if loss of *Kif3a* in various tissues of the craniofacial complex impacted cell proliferation and cell death, we performed immunostaining for phosphohistone H3 (PHH3) and cleaved caspase 3 (CC3) in the developing frontonasal prominence and palate at e11.5 (see areas analyzed for quantification in S3A and S3B Fig). We found that loss of *Kif3a* had tissue specific effects on cell proliferation and cell death (Table 2, S1 Table). Loss of *Kif3a* within NCCs of the frontonasal prominence caused a significant decrease in cell proliferation, whereas loss of *Kif3a* in surface ectoderm or a combination of NCCs, surface ectoderm and neuroectoderm did not cause a significant change in proliferation relative to wild-type embryos (Fig 2I–2L and 2Z). Furthermore, loss of *Kif3a* in the surface ectoderm significantly increased the amount of cell death within the frontonasal prominence, whereas cell death was not significantly impacted when *Kif3a* was lost in NCCs alone (*Wnt1-Cre*), or a combination of tissues (*AP2-Cre*) (Fig 2M–2P and 2AA). In the developing palatal shelves, loss of *Kif3a* did not cause a statistically significant change in cell proliferation (Fig 2Q–2T and 2BB); however, a significant increase in cell death was observed within the palatal shelves of *Kif3a*^*f/f*^*;Crect* and *Kif3a*^*f/f*^*;AP2-Cre* mutants (Fig 2U–2X and 2CC). Thus, taken together, these results suggest a tissue specific function for *Kif3a* and the cilium in the developing craniofacial complex.

**Table 2:** Summary of measured values for cell proliferation and cell death in the frontonasal prominence and palatal shelves in e11.5 *Kif3a* mutants.

Genotype	Stage	Values	PHH3 FNP	CC3 FNP	PHH3 PS	CC3 PS
Control	e11.5	n’s*	15	14	10	12
		Average^+^	69.73	4.93	113.27	12.57
		STD	26.94	3.46	16.92	5.65
		P-value	N/A	N/A	N/A	N/A
*Kif3a*^*f/f*^*;Wnt1-Cre*	e11.5	n’s*	12	17	20	20
		Average^+^	41.63	4.78	115.06	46.64
		STD	15.13	4.78	35.55	8.07
		P-value	0.002	0.920	0.853	0.767
*Kif3a*^*f/f*^*; Crect*	e11.5	n’s*	12	12	12	13
		Average^+^	53.64	21.77	116.12	37.29
		STD	11.68	10.88	24.89	11.26
		P-value	0.050	0.0002	0.747	1.5x10^-6^
*Kif3a*^*f/f*^*;AP2-Cre*	e11.5	n’s*	19	13	17	16
		Average^+^	58.19	6.05	100.40	20.77
		STD	25.30	4.24	25.97	11.98
		P-value	0.208	0.463	0.132	0.021

frontonasal prominence (FNP), palatal shelves (PS)

* n values indicate number of sections analyzed

^+^ all averages represent the number of PHH3/CC3 positive cells normalized over either FNP or PS area

**Fig 3:**
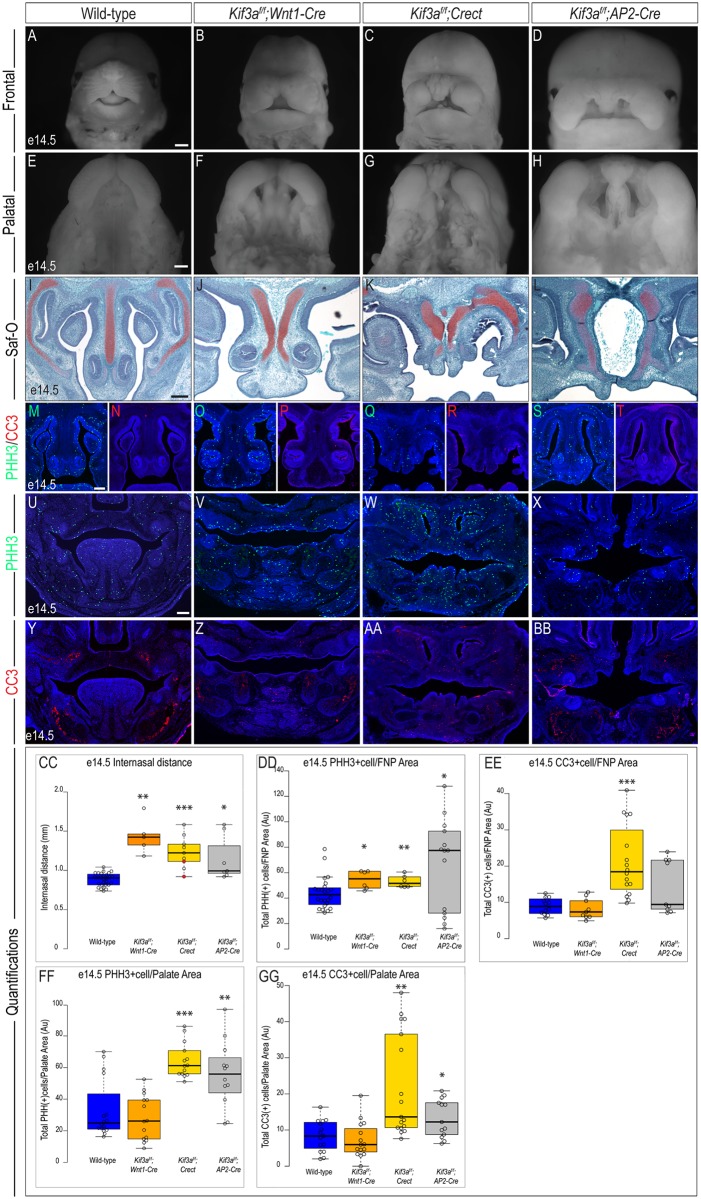
Analysis of e14.5 *Kif3a* conditional mutants. Frontal and palatal views of e14.5 (A, E) wild-type, (B, F) *Kif3a*^*f/f*^*;Wnt1-Cre*, (C, G) *Kif3a*^*f/f*^*;Crect*, (D, H) *Kif3a*^*f/f*^*;AP2-Cre* embryos. Safranin-O staining on frontal sections of e14.5 (I) wild-type, (J) *Kif3a*^*f/f*^*;Wnt1-Cre*, (K) *Kif3a*^*f/f*^*;Crect*, (L) *Kif3a*^*f/f*^*;AP2-Cre* embryos. PHH3 staining in e14.5 frontonasal sections of (M) wild-type (n = 24), (O) *Kif3a*^*f/f*^*;Wnt1-Cre* (n = 6), (Q) *Kif3a*^*f/f*^*;Crect* (n = 6), (Q) *Kif3a*^*f/f*^*;AP2-Cre* (n = 14) embryos. CC3 staining in e14.5 frontonasal sections of (N) wild-type (n = 12), (P) *Kif3a*^*f/f*^*;Wnt1-Cre* (n = 9); (R) *Kif3a*^*f/f*^*;Crect* (n = 17), (T) *Kif3a*^*f/f*^*;AP2-Cre* (n = 9) embryos. PHH3 staining in e14.5 palatal sections of (U) wild-type (n = 16), (V) *Kif3a*^*f/f*^*;Wnt1-Cre* (n = 14), (W) *Kif3a*^*f/f*^*;Crect* (n = 14), (X) *Kif3a*^*f/f*^*;AP2-Cre* (n = 12) embryos. CC3 staining in e14.5 palatal sections of (Y) wild-type (n = 15), (Z) *Kif3a*^*f/f*^*;Wnt1-Cre* (n = 15); (AA) *Kif3a*^*f/f*^*;Crect* (n = 17), (BB) *Kif3a*^*f/f*^*;AP2-Cre* (n = 13) embryos. (CC-GG) Quantification of measured values represented as box plots where the median and interquartile range are displayed. Red dots on the graph represent *Kif3a*^*f/f*^*;Crect* mutants that did not display a craniofacial phenotype and were not included in the rest of the analyses (2/9). For each genotype, *n = 3* embryos were utilized for PHH3/CC3 analyses. P-values: (*) 0.05≥P>0.01; (**) 0.01≥P>0.001; (***) P≤0.001. Scale bars: (A-D) 375 μm, (E-H) 500 μm, (I-L) 150 μm, and (M-BB) 250 μm.

We continued our analysis of these tissue-specific mutants at e14.5. Again, the most striking phenotype of *Kif3a*^*f/f*^;*Wnt1-Cre* embryos was the severe midline widening (Fig 3A, 3B and 3CC; n = 5). The midline of *Kif3a*^*f/f*^;*Crect* embryos was also significantly wider, highly dysmorphic and featured numerous tissue nodules (Fig 3C and 3CC; n = 9). Similarly, *Kif3a*^*f/f*^;*AP2-Cre* embryos had a significantly widened midline with a dysmorphic frontonasal prominence (Fig 3D and 3CC; n = 7). Despite an overall significant increase of internasal width, we observed some variability in the severity of the midfacial phenotype in *Kif3a*^*f/f*^;*AP2-Cre* embryos (S4A and S4B Fig). All three mutants also presented with cleft palate (Fig 3E–3H). We also examined the development of the mandibular prominence within these three mutants. Consistent with our previous reports, *Kif3a*^*f/f*^;*Wnt1-Cre* embryos had micrognathia (undersized jaw) and aglossia (no tongue) (S5A and S5B Fig). In contrast, the tongue was clearly present in *Kif3a*^*f/f*^;*Crect* embryos, yet there were tissue hyperplasias similar to those seen on the developing frontonasal prominence (S5C Fig). The developing mandible of *Kif3a*^*f/f*^;*AP2-Cre* embryos resembled that of the *Kif3a*^*f/f*^;*Wnt1-Cre* embryos, presenting with aglossia and mild micrognathia (S5D Fig).

Histologically, the midfacial widening in *Kif3a*^*f/f*^;*Wnt1-Cre* embryos was apparent by the presence of a duplicated of the nasal septum (Fig 3I and 3J) [6]. The nasal septum is a cartilaginous structure derived from NCCs that occupy the frontonasal prominence. Both *Kif3a*^*f/f*^;*Crect* and *Kif3a*^*f/f*^;*AP2-Cre* embryos presented with a duplicated nasal septum (Fig 3K and 3L), yet with varying degrees of penetrance and severity (Table 3 and S4D and S4E Fig). We next analyzed each mutant to determine if cell proliferation and cell death were aberrant in either the developing frontonasal prominence or palate (see areas analyzed for quantification in S3C and S3D Fig). Cell proliferation was significantly increased in the developing frontonasal prominence in all three mutants (Fig 3M, 3O, 3Q, 3S and 3DD; Table 3); however, we also observed a significant increase in cell death in *Kif3a*^*f/f*^*;Crect* mutants (Fig 3N, 3P, 3R, 3T and 3EE). We next examined how loss of *Kif3a* affected palatal development at e14.5 in all three mutants. Neither cell proliferation nor cell death was significantly altered in the palate of *Kif3a*^*fl/fl*^*;Wnt1-Cre* mutants (Fig 3U, 3V, 3Y, 3Z, 3FF and 3GG). Cell proliferation and cell death were, however; significantly increased in *Kif3a*^*f/f*^*;Crect* and *Kif3a*^*f/f*^*;AP2-Cre* mutants (Fig 3W, 3X, 3AA, 3BB, 3FF and 3GG). Taken as a whole, these data again suggest that *Kif3a* and cilia in different craniofacial tissues have distinct roles in regulating craniofacial development, as each Cre-driver resulted in a unique phenotype and alterations in cellular behaviors. We next set out to determine if these phenotypes were specific to *Kif3a*.

**Table 3:** Summary of measured values for cell proliferation and cell death in the frontonasal prominence and palatal shelves in e14.5 *Kif3a* mutants.

Genotype	Stage	Values	PHH3 FNP	CC3 FNP	PHH3 PS	CC3 PS
Control	e14.5	n’s*	24	12	16	15
		Average^+^	44.08	8.94	33.07	8.38
		STD	12.28	2.26	18.64	4.31
		P-value	N/A	N/A	N/A	N/A
*Kif3a*^*f/f*^*;Wnt1-Cre*	e14.5	n’s*	6	9	14	15
		Average^+^	54.36	10.33	28.26	7.22
		STD	7.23	4.88	14.36	5.04
		P-value	0.020	0.386	0.430	0.505
*Kif3a*^*f/f*^*; Crect*	e14.5	n’s*	6	17	14	17
		Average^+^	53.09	21.23	67.23	22.10
		STD	4.81	9.90	15.43	14.29
		P-value	0.010	0.0002	5.1x10^-6^	0.001
*Kif3a*^*f/f*^*;AP2-Cre*	e14.5	n’s*	14	9	12	13
		Average^+^	66.35	14.36	55.74	13.35
		STD	36.01	7.36	21.01	5.28
		P-value	0.041	0.061	0.007	0.013

frontonasal prominence (FNP), palatal shelves (PS)

* n values indicate number of sections analyzed

^+^ all averages represent the number of PHH3/CC3 positive cells normalized over either FNP or PS area

### Loss of *Ift88* in tissue-specific domains of the craniofacial complex phenocopies *Kif3a* mutants

**Fig 4:**
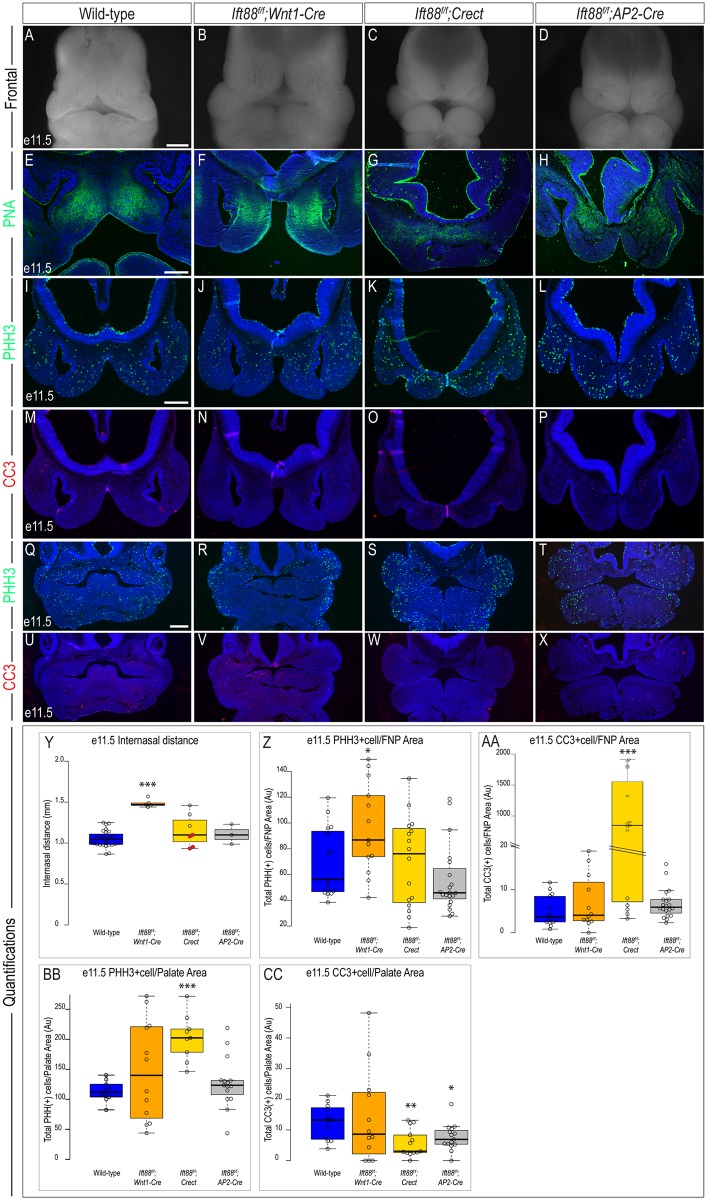
Analysis of e11.5 *Ift88* conditional mutants. Frontal view of e11.5 (A) wild-type, (B) *Ift88*^*f/f*^*;Wnt1-Cre*, (C) *Ift88*^*f/f*^*;Crect*, and (D) *Ift88*^*f/f*^*;AP2-Cre* embryos. PNA staining in e11.5 sections of (E) wild-type, (F) *Ift88*^*f/f*^*;Wnt1-Cre*, (G) *Ift88*^*f/f*^*;Crect*, (H) *Ift88*^*f/f*^*;AP2-Cre* embryos. PHH3 staining in e11.5 frontonasal sections of (I) wild-type (n = 15), (J) *Ift88*^*f/f*^*;Wnt1-Cre* (n = 13), (K) *Ift88*^*f/f*^*;Crect* (n = 16), (L) *Ift88*^*f/f*^*;AP2-Cre* (n = 20) embryos. CC3 staining in e11.5 frontonasal sections of (M) wild-type (n = 14), (N) *Ift88*^*f/f*^*;Wnt1-Cre* (n = 12); (O) *Ift88*^*f/f*^*;Crect* (n = 15), (P) *Ift88*^*f/f*^*;AP2-Cre* (n = 20) embryos. PHH3 staining in e11.5 palatal sections of (Q) wild-type (n = 10), (R) *Ift88*^*f/f*^*;Wnt1-Cre* (n = 12), (S) *Ift88*^*f/f*^*;Crect* (n = 9), (T) *Ift88*^*f/f*^*;AP2-Cre* (n = 15) embryos. CC3 staining in e11.5 palatal sections of (U) wild-type (n = 12), (V) *Ift88*^*f/f*^*;Wnt1-Cre* (n = 12); (W) *Ift88*^*f/f*^*;Crect* (n = 13), (X) *Ift88*^*f/f*^*;AP2-Cre* (n = 15) embryos. (Y-CC) Quantification of measured values represented as box plots where median and interquartile range are displayed. Red dots on the graph represent *Ift88*^*f/f*^*;Crect* mutants that did not display a craniofacial phenotype and were not included in the rest of the analyses (4/7). For each genotype, *n = 3* embryos were utilized for PHH3/CC3 analyses. P-values: (*) 0.05≥P>0.01; (**) 0.01≥P>0.001; (***) P≤0.001. Scale bars: (A-D) 500 μm, (E-H) 200 μm, (I-P) 225 μm and (Q-X) 350μm.

IFT88 is another anterograde IFT protein essential for ciliogenesis [32]. To determine if the craniofacial phenotypes we observed with ablation of *Kif3a* were specific to *Kif3a* itself, or due to impaired ciliogenesis, we repeated our previous approach and conditionally knocked out *Ift88* with the same drivers used to conditionally ablate *Kif3a*. As mentioned above, the first distinguishing feature of *Kif3a*^*f/f*^;*Wnt1-Cre* embryos was a widened midline at e11.5, as determined by internasal distance [6, 33]. *Ift88*^*f/f*^;*Wnt1-Cre* embryos at e11.5 also had a significant increase in the internasal distance (Fig 4A, 4B and 4Y; n = 5), albeit slightly less severe than that observed in *Kif3a*^*f/f*^;*Wnt1-Cre* embryos (Fig 2, Tables 2 and 4). Interestingly, neither *Ift88*^*f/f*^;*Crect* (n = 7) nor *Ift88*^*f/f*^;*AP2-Cre* (n = 3) embryos presented with significant midfacial widening relative to wild-type embryos (Fig 4C, 4D and 4Y). Despite the lack of midfacial widening, *Ift88*^*f/f*^;*AP2-Cre* embryos did have medially rotated nasal pits, similar to those observed in *Kif3a*^*f/f*^;*AP2-Cre* mutants (compare Figs 2D and 4D). Thus, while *Ift88* mutants did not perfectly parallel *Kif3a* mutants, a significant overlap in craniofacial phenotypes was observed.

**Table 4:** Summary of measured values for cell proliferation and cell death in the frontonasal prominence and palatal shelves in e11.5 *Ift88* mutants.

Genotype	Stage	Values	PHH3 FNP	CC3 FNP	PHH3 PS	CC3 PS
Control	e11.5	n’s*	15	14	10	12
		Average^+^	69.73	4.93	113.27	12.57
		STD	26.94	3.46	16.92	5.65
		P-value	N/A	N/A	N/A	N/A
*Ift88*^*f/f*^*;Wnt1-Cre*	e11.5	n’s*	13	12	12	12
		Average^+^	95.77	6.95	147.84	14.13
		STD	35.04	6.23	83.00	15.12
		P-value	0.040	0.331	0.184	0.743
*Ift88*^*f/f*^*; Crect*	e11.5	n’s*	16	15	9	13
		Average^+^	69.85	811.18	203.15	5.74
		STD	34.76	753.00	38.19	4.47
		P-value	0.991	0.0007	4.8x10^-5^	0.003
*Ift88*^*f/f*^*;AP2-Cre*	e11.5	n’s*	20	20	15	15
		Average^+^	55.61	6.72	127.98	7.63
		STD	26.25	3.42	42.60	4.27
		P-value	0.129	0.145	0.243	0.021

frontonasal prominence (FNP), palatal shelves (PS)

* n values indicate number of sections analyzed

^+^ all averages represent the number of PHH3/CC3 positive cells normalized over either FNP or PS area

While the overall morphology of the mutants appeared extremely similar, we again examined cell differentiation, cell proliferation and cell death of *Ift88* tissue-specific mutants. We examined the earliest stages of cell differentiation and found that, similar to *Kif3a* mutants, *Ift88* mutants contained altered domains of PNA positive cells (Fig 4E–4H). We next examined cell proliferation and cell death. Loss of *Ift88* within NCCs (*Wnt1-Cre*) of the frontonasal prominence caused a significant increase in cell proliferation, whereas loss of *Ift88* in surface ectoderm (*Crect*) or a combination of NCCs, surface ectoderm and neuroectoderm (*AP2-Cre*) did not cause a significant change in proliferation relative to wild-type embryos at e11.5 (Fig 4I–4L and 4Z). Furthermore, the loss of *Ift88* in surface ectoderm (*Crect*) significantly increased the amount of cell death within the frontonasal prominence, whereas cell death was not significantly impacted when *Ift88* was lost in NCCs alone (*Wnt1-Cre*), or a combination of tissues (*AP2-Cre*) (Fig 4M–4P and 4AA). Loss of *Ift88* in surface ectoderm (*Crect*) significantly increased cell proliferation in the developing palatal shelves, yet there was not a significant change in proliferation when *Ift88* was ablated from NCCs (*Wnt1-Cre*) or a combination of tissues (*AP2-Cre*) (Fig 4Q–4T and 4BB). Cell death was also affected in a tissue specific manner. Whereas loss of *Ift88* in NCCs (*Wnt1-Cre*) did not affect cell death in the developing palate, loss of *Ift88* in surface ectoderm (*Crect*) and a combination of craniofacial tissues (*AP2-Cre*) resulted in a significant decrease in cell death (Fig 4U–4X and 4CC). Thus, while the gross phenotypes of *Kif3a* and *Ift88* mutants are similar, these results suggest a tissue specific function for *Ift88*, distinct from *Kif3a*.

**Fig 5:**
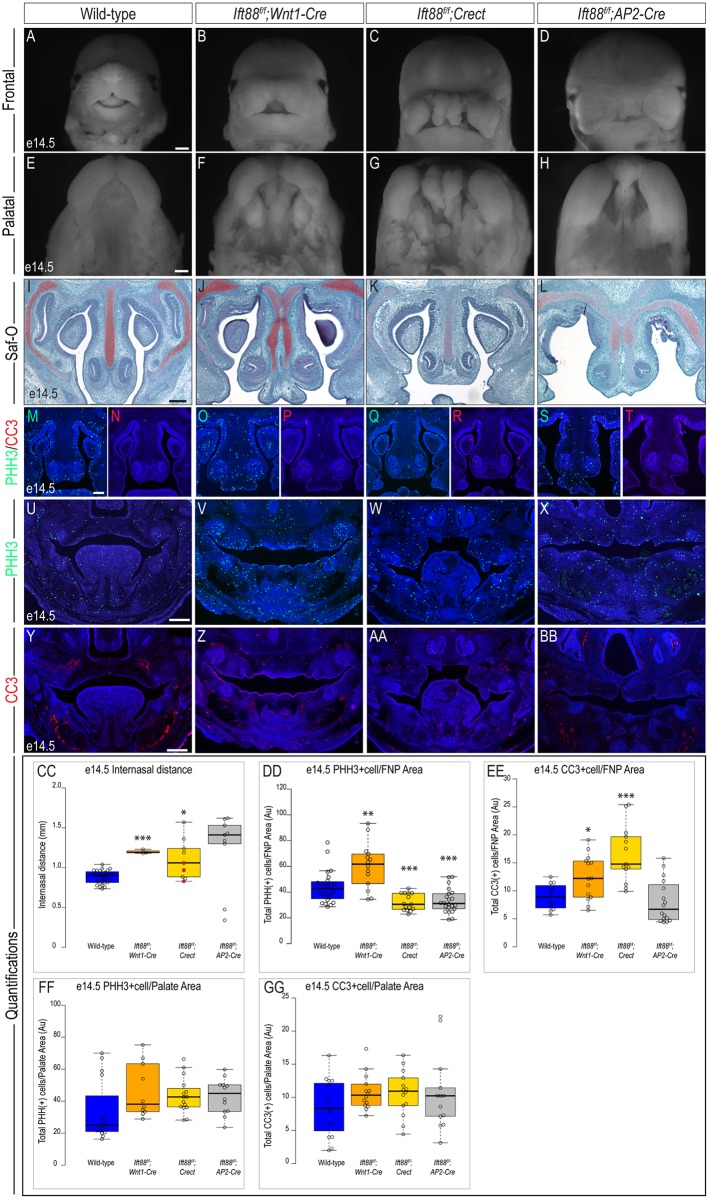
Analysis of e14.5 *Ift88* conditional mutants. Frontal and palatal views of e14.5 (A, E) wild-type, (B, F) *Ift88*^*f/f*^*;Wnt1-Cre*, (C, G) *Ift88*^*f/f*^*;Crect*, (D, H) *Ift88*^*f/f*^*;AP2-Cre* embryos. Safranin-O staining on frontal sections of e14.5 (I) wild-type, (J) *Ift88*^*f/f*^*;Wnt1-Cre*, (K) *Ift88*^*f/f*^*;Crect*, (L) *Ift88*^*f/f*^*;AP2-Cre* embryos. PHH3 staining in e14.5 frontonasal sections of (M) wild-type (n = 24), (O) *Ift88*^*f/f*^*;Wnt1-Cre* (n = 14), (Q) *Ift88*^*f/f*^*;Crect* (n = 15), (S) *Ift88*^*f/f*^*;AP2-Cre* (n = 22) embryos. CC3 staining in e14.5 frontonasal sections of (N) wild-type (n = 12), (P) *Ift88*^*f/f*^*;Wnt1-Cre* (n = 14); (R) *Ift88*^*f/f*^*;Crect* (n = 13), (T) *Ift88*^*f/f*^*;AP2-Cre* (n = 16) embryos. PHH3 staining in e14.5 palatal sections of (U) wild-type (n = 16), (V) *Ift88*^*f/f*^*;Wnt1-Cre* (n = 10), (W) *Ift88*^*f/f*^*;Crect* (n = 15), (X) *Ift88*^*f/f*^*;AP2-Cre* (n = 12) embryos. CC3 staining in e14.5 palatal sections of (Y) wild-type (n = 15), (Z) *Ift88*^*f/f*^*;Wnt1-Cre* (n = 14); (AA) *Ift88*^*f/f*^*;Crect* (n = 14), (BB) *Ift88*^*f/f*^*;AP2-Cre* (n = 13) embryos. (CC-GG) Quantification of measured values represented as box plots where the median and interquartile range are displayed. Red dots on the graph represent *Ift88*^*f/f*^*;Crect* mutants that did not display a craniofacial phenotype and were not included in the rest of the analyses (3/9). For each genotype, *n = 3* embryos were utilized for PHH3/CC3 analyses. P-values: (*) 0.05≥P>0.01; (**) 0.01≥P>0.001; (***) P≤0.001. Scale bars: (A-D) 375 μm, (E-H) 500 μm, (I-L) 150 μm, and (M-BB) 250 μm.

The conservation in craniofacial phenotypes between mutants generated from both a conditional loss of *Kif3a* and *Ift88* continued at e14.5. The characteristic midline widening of *Kif3a*^*f/f*^;*Wnt1-Cre* was recapitulated in *Ift88*^*f/f*^;*Wnt1-Cre* embryos, albeit not as severe (Fig 5A, 5B and 5CC; n = 3). Similar to *Kif3a*^*f/f*^;*Crect* embryos, *Ift88*^*f/f*^;*Crect* embryos had a significantly wider midface that was both highly dysmorphic and hyperplasic with numerous tissue nodules (Fig 5C and 5CC; n = 9). As observed with the *Kif3a*^*f/f*^;*AP2-Cre* embryos, *Ift88*^*f/f*^;*AP2-Cre* embryos at e14.5 had a combination of phenotypes from the *Ift88*^*f/f*^;*Wnt1-Cre* and *Ift88*^*f/f*^;*Crect* embryos: a widened midline with a dysmorphic and hyperplastic FNP (Fig 5D and 5CC; n = 9). The internasal distance did not measure as significantly different in *Ift88*^*f/f*^;*AP2-Cre* mutants; however, this is likely due to the large range in internasal distances measured in *Ift88*^*f/f*^;*AP2-Cre* embryos (S4C and S4F Fig). The midfacial phenotypes were accompanied by cleft palate. Similar to their *Kif3a* counterparts, all three *Ift88*^*f/f*^ mutants had cleft palate (Fig 5E–5H). We next examined the development of the mandibular prominence in these mutants. *Ift88*^*f/f*^;*Wnt1-Cre* embryos also displayed aglossia and micrognathia (S5E–S5G Fig). The tongue was clearly present in *Ift88*^*f/f*^;*Crect* embryos, yet as in *Kif3a*^*f/f*^;*Crect* embryos, there were tissue hyperplasias similar to those seen on the developing frontonasal prominence (S5F Fig). The developing mandible of *Ift88*^*f/f*^;*AP2-Cre* embryos resembled that of the *Kif3a*^*f/f*^;*AP2-Cre* embryos, presenting with aglossia and mild micrognathia (S5G Fig).

The characteristic duplication of the nasal septum was again present in the *Ift88*^*f/f*^;*Wnt1-Cre* embryos (Fig 5I and 5J). We did not observe a duplicated nasal septum in *Ift88a*^*f/f*^;*Crect* embryos (Fig 5K), whereas *Ift88*^*f/f*^;*AP2-Cre* embryos did have a duplicated septum (Fig 5L). We again examined cell proliferation and cell death within this area. Within the frontonasal prominence, cell proliferation was significantly increased in *Ift88*^*f/f*^;*Wnt1-Cre* embryos (Fig 5M, 5O and 5DD; Table 5), similar to that observed in *Kif3a*^*f/f*^;*Wnt1-Cre* embryos. Conversely, we observed *Ift88*^*f/f*^;*Crect* and *Ift88*^*f/f*^*;AP2-Cre* embryos had a slight, but significant decrease in proliferation within the frontonasal prominence (Fig 5Q, 5S and 5DD). With respect to cell death, we observed a significant increase in CC3-postive cells in both *Ift88*^*f/f*^;*Wnt1-Cre* and *Ift88*^*fl/fl*^*;Crect* embryos (Fig 5N, 5P, 5R and 5EE). No significant change between the number of CC3-positive cells was detected in *Ift88*^*fl/fl*^*;AP2-Cre* embryos relative to control embryos (Fig 5T and 5EE). Interestingly, there was no significant change in cell proliferation or cell death in any of the *Ift88* mutants within the developing palate at e14.5 (Fig 5U–5BB, 5FF and 5GG). In sum, the gross craniofacial phenotypes between *Kif3a* and *Ift88* mutants were relatively conserved, despite some differences in cell behaviors in affected areas. Together these data further supported the hypothesis that the cilium has distinct roles in individual tissues of the craniofacial complex, while additionally suggesting that ciliary proteins themselves may also have specific functions within each tissue.

**Table 5:** Summary of measured values for cell proliferation and cell death in the frontonasal prominence and palatal shelves in e14.5 *Ift88* mutants.

Genotype	Stage	Values	PHH3 FNP	CC3 FNP	PHH3 PS	CC3 PS
Control	e14.5	n’s*	24	12	16	15
		Average^+^	44.08	8.94	33.07	8.38
		STD	12.28	2.26	18.64	4.31
		P-value	N/A	N/A	N/A	N/A
*Ift88*^*f/f*^*;Wnt1-Cre*	e14.5	n’s*	14	14	10	14
		Average^+^	60.45	12.40	46.48	10.78
		STD	17.78	3.97	16.87	2.70
		P-value	0.0064	0.011	0.072	0.082
*Ift88*^*f/f*^*; Crect*	e14.5	n’s*	15	13	15	14
		Average^+^	32.10	16.59	43.59	10.63
		STD	6.53	5.11	11.14	3.43
		P-value	0.0004	0.0001	0.065	0.130
*Ift88*^*f/f*^*;AP2-Cre*	e14.5	n’s*	22	16	12	13
		Average^+^	33.05	8.08	42.98	10.62
		STD	9.28	3.84	11.12	5.77
		P-value	0.001	0.464	0.091	0.262

frontonasal prominence (FNP), palatal shelves (PS)

* n values indicate number of sections analyzed

^+^ all averages represent the number of PHH3/CC3 positive cells normalized over either FNP or PS area

### Loss of *Ttc21b* in tissue-specific domains of the craniofacial complex does not phenocopy *Kif3a* or *Ift88* mutants

KIF3A and IFT88 are both anterograde intraflagellar transport proteins that function in the IFT-B complex to facilitate the transport of molecular cargo from the cell body to ciliary tip. TTC21B (also known as *Ift139* and *Thm1*) is a retrograde intraflagellar transport protein that functions in the IFT-A complex in the retrograde transport of molecular cargo from the ciliary tip to the cell body [13]. To determine if ciliary proteins that function in distinct areas of the cilium affect craniofacial development differentially, we repeated our experimental strategy with *Ttc21b*^*f/aln*^ mice, which have one floxed allele and one allele that contains the *alien* mutation, a null allele of *Ttc21b* [13].

**Fig 6:**
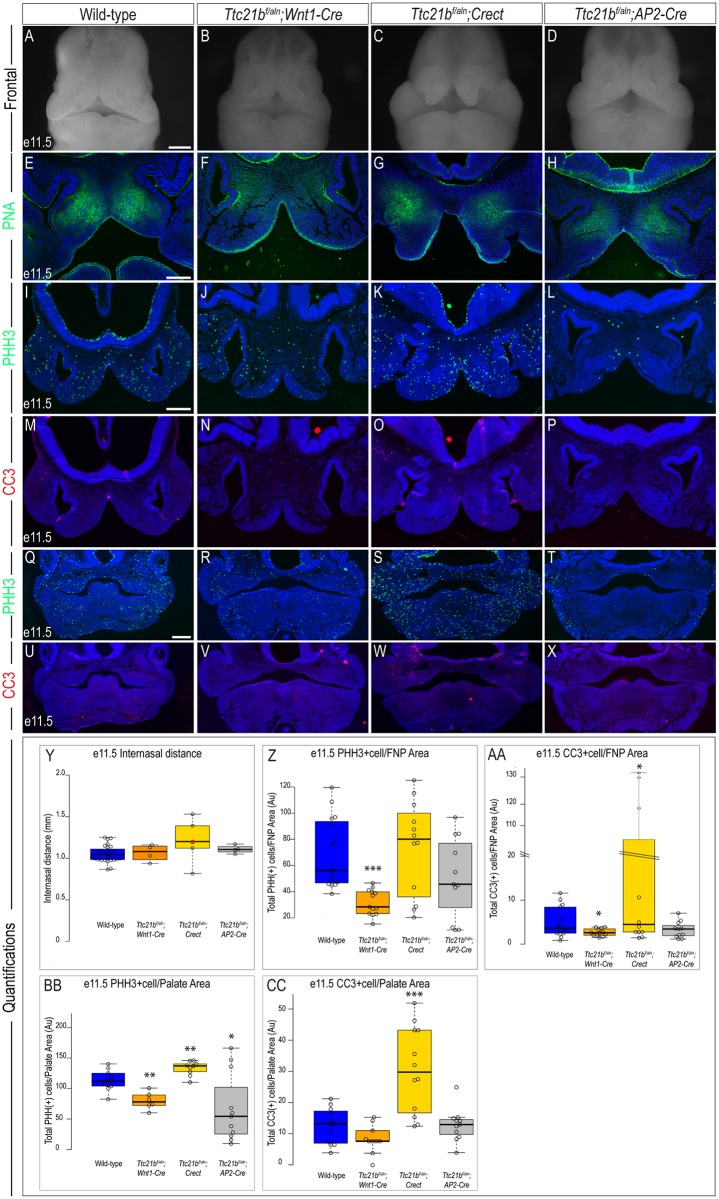
Analysis of e11.5 *Ttc21b* conditional mutants. Frontal view of e11.5 (A) wild-type, (B) *Ttc21b*^*f/aln*^*;Wnt1-Cre*, (C) *Ttc21b*^*f/aln*^*;Crect*, and (D) *Ttc21b*^*f/aln*^*;AP2-Cre* embryos. PNA staining in e11.5 sections of (E) wild-type, (F) *Ttc21b*^*f/aln*^*;Wnt1-Cre*, (G) *Ttc21b*^*f/aln*^*;Crect*, (H) *Ttc21b*^*f/aln*^*;AP2-Cre* embryos. PHH3 staining in e11.5 frontonasal sections of (I) wild-type (n = 15), (J) *Ttc21b*^*f/aln*^*;Wnt1-Cre* (n = 13), (K) *Ttc21b*^*f/aln*^*;Crect* (n = 12), (L) *Ttc21b*^*f/aln*^*;AP2-Cre* (n = 11) embryos. CC3 staining in e11.5 frontonasal sections of (M) wild-type (n = 14), (N) *Ttc21b*^*f/aln*^*;Wnt1-Cre* (n = 12), (O) *Ttc21b*^*f/aln*^*;Crect* (n = 12), (P) *Ttc21b*^*f/aln*^*;AP2-Cre* (n = 11) embryos. PHH3 staining in e11.5 palatal sections of (Q) wild-type (n = 10), (R) *Ttc21b*^*f/aln*^*;Wnt1-Cre* (n = 6), (S) *Ttc21b*^*f/aln*^*;Crect* (n = 9), (T) *Ttc21b*^*f/aln*^*;AP2-Cre* (n = 11) embryos. CC3 staining in e11.5 palatal sections of (U) wild-type (n = 12), (V) *Ttc21b*^*f/aln*^*;Wnt1-Cre* (n = 10), (W) *Ttc21b*^*f/aln*^*;Crect* (n = 12), (X) *Ttc21b*^*f/aln*^*;AP2-Cre* (n = 11) embryos. (Y-CC) Quantification of measured values represented as box plots where the median and interquartile range are displayed. Red dots on the graph represent *Ttc21b*^*f/aln*^*;Crect* mutants that did not display a craniofacial phenotype and were not included in the rest of the analyses (1/5). For each genotype, *n = 3* embryos were utilized for PHH3/CC3 analyses. P-values: (*) 0.05≥P>0.01; (**) 0.01≥P>0.001; (***) P≤0.001. Scale bars: (A-D) 500 μm, (E-H) 200 μm, (I-P) 225 μm, and (Q-X) 350μm.

*Kif3a*^*f/f*^*;Wnt1-Cre* and *Ift88*^*f/f*^;*Wnt1-Cre* embryos at e11.5 have significant midfacial defects characterized by an increase in the internasal distance (Figs 2 & 4). In contrast, *Ttc21b*^*f/aln*^*;Wnt1-Cre* embryos do not have a significant difference in internasal distance when compared to wild-type embryos (Fig 6A, 6B and 6Y; n = 4). Similar to *Kif3a*^*f/f*^*;Crect* and *Ift88*^*f/f*^;*Crect* mutants, *Ttc21b*^*f/aln*^*;Crect* embryos did not have a significantly wider internasal distance, yet their nasal pits were patent due to a failure of fusion between the frontonasal, lateral nasal and maxillary prominences (Fig 6C and 6Y; n = 5). *Ttc21b*^*f/aln*^;*AP2-Cre* embryos, in which neural crest, surface ectoderm and some neuroectoderm were affected, also did not display midfacial widening (Fig 6D and 6Y; n = 4). These results again supported a hypothesis that the cilium plays tissue specific roles in craniofacial development. Furthermore, the observation of distinctly different phenotypes occurring with the same drivers suggested that individual ciliary genes have a unique function in each tissue.

We next analyzed each mutant to determine if loss of *Ttc21b* in different tissues and tissue combinations had an effect on cell differentiation, cell proliferation and cell death. Once again, differentiation of early skeletal condensations was examined via PNA staining. Despite the pattern of the early condensations being different, PNA positive domains were still detected in all mutants (Fig 6E–6H). To determine if cellular processes including cell proliferation and cell death were altered in these mutants, we examined PHH3 and CC3 staining in the developing frontonasal prominence and palate. Cell proliferation was significantly reduced in the frontonasal prominence of *Ttc21b*^*f/aln*^*;Wnt1-Cre* mutants, yet there was no significant change in proliferation within *Ttc21b*^*f/aln*^*;Crect* or *Ttc21b*^*f/aln*^*;AP2-Cre* mutants (Fig 6I–6L and 6Z; Table 6). Cell death was also altered in a tissue-specific manner. There was a significant reduction in cell death within the frontonasal prominence of *Ttc21b*^*f/aln*^*;Wnt1-Cre* mutants, a significant increase in cell death in *Ttc21b*^*f/aln*^*;Crect* mutants and no change cell death in *Ttc21b*^*f/aln*^*;AP2-Cre* mutants relative to wild-type embryos (Fig 6M–6P and 6AA). There were also significant changes in cell proliferation and cell death within the developing palate. Loss of *Ttc21b* in NCCs (*Wnt1-Cre*) caused a significant reduction in cell proliferation, loss of *Ttc21b* in surface ectoderm (*Crect*) caused a significant increase in cell proliferation, and loss of *Ttc21b* in NCCs, surface ectoderm and neuroectoderm (*AP2-Cre*) caused a significant reduction in proliferation (Fig 6Q–6T and 6BB). Finally, we examined how loss of *Ttc21b* in various tissues affected cell death within the developing palate. Whereas loss of *Ttc21b* in NCCs (*Wnt1-Cre)* or a combination of NCCs, surface ectoderm and neuroectoderm (*AP2-Cre*) had no effect on cell death in the developing palate, *Ttc21b*^*f/aln*^*;Crect* embryos had a significant increase in cell death relative to wild-type controls (Fig 6U–6X and 6CC).

**Table 6:** Summary of measured values for cell proliferation and cell death in the frontonasal prominence and palatal shelves in e11.5 *Ttc21b* mutants.

Genotype	Stage	Values	PHH3 FNP	CC3 FNP	PHH3 PS	CC3 PS
Control	e11.5	n’s*	15	14	10	12
		Average^+^	69.73	4.93	113.27	12.57
		STD	26.94	3.46	16.92	5.65
		P-value	N/A	N/A	N/A	N/A
*Ttc21b*^*f/aln*^*;Wnt1-Cre*	e11.5	n’s*	13	12	6	10
		Average^+^	31.93	2.70	80.25	8.37
		STD	9.78	0.84	14.17	4.52
		P-value	7.9x10^-5^	0.034	0.001	0.072
*Ttc21b*^*f/aln*^*; Crect*	e11.5	n’s*	12	12	9	12
		Average^+^	73.77	41.78	133.91	30.58
		STD	35.87	56.85	11.77	13.83
		P-value	0.750	0.046	0.007	0.001
*Ttc21b*^*f/aln*^*;AP2-Cre*	e11.5	n’s*	11	11	11	11
		Average^+^	50.87	3.32	68.37	12.88
		STD	30.78	1.87	56.00	5.29
		P-value	0.120	0.152	0.026	0.896

frontonasal prominence (FNP), palatal shelves (PS)

* n values indicate number of sections analyzed

^+^ all averages represent the number of PHH3/CC3 positive cells normalized over either FNP or PS area

**Fig 7:**
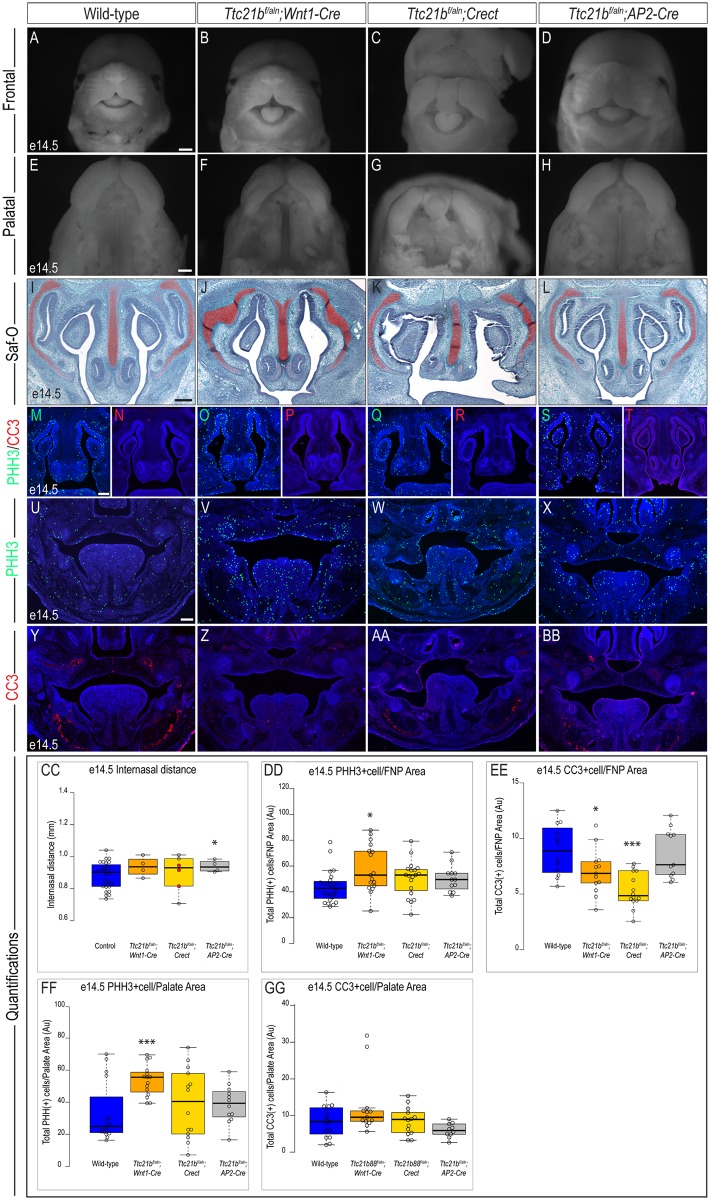
Analysis of e14.5 *Ttc21b* conditional mutants. Frontal and palatal views of e14.5 (A, E) wild-type, (B, F) *Ttc21b*^*f/aln*^*;Wnt1-Cre*, (C, G) *Ttc21b*^*f/aln*^*;Crect*, (D, H) *Ttc21b*^*f/aln*^*;AP2-Cre* embryos. Safranin-O staining on frontal sections of e14.5 (I) wild-type, (J) *Ttc21b*^*f/aln*^*;Wnt1-Cre*, (K) *Ttc21b*^*f/aln*^*;Crect*, (L) *Ttc21b*^*f/aln*^*;AP2-Cre* embryos. PHH3 staining in e14.5 frontonasal sections of (M) wild-type (n = 24), (O) *Ttc21b*^*f/aln*^*;Wnt1-Cre* (n = 17), (Q) *Ttc21b*^*f/aln*^*;Crect* (n = 19), (S) *Ttc21b*^*f/aln*^*;AP2-Cre* (n = 12) embryos. CC3 staining in e14.5 frontonasal sections of (N) wild-type (n = 12), (P) *Ttc21b*^*f/aln*^*;Wnt1-Cre* (n = 13); (R) *Ttc21b*^*f/aln*^*;Crect* (n = 14), (T) *Ttc21b*^*f/aln*^*;AP2-Cre* (n = 11) embryos. PHH3 staining in e14.5 palatal sections of (U) wild-type (n = 16), (V) *Ttc21b*^*f/aln*^*;Wnt1-Cre* (n = 15), (W) *Ttc21b*^*f/aln*^*;Crect* (n = 14), (X) *Ttc21b*^*f/aln*^*;AP2-Cre* (n = 12) embryos. CC3 staining in e14.5 palatal sections of (Y) wild-type (n = 15), (Z) *Ttc21b*^*f/aln*^*;Wnt1-Cre* (n = 13); (AA) *Ttc21b*^*f/aln*^*;Crect* (n = 14), (BB) *Ttc21b*^*f/aln*^*;AP2-Cre* (n = 10) embryos. (CC-GG) Quantification of measured values represented as box plots where the median and interquartile range are displayed. Red dots on the graph represent *Ttc21b*^*f/aln*^*;Crect* mutants that did not display a craniofacial phenotype and were not included in the rest of the analysis (3/6). For each genotype, *n = 3* embryos were utilized for PHH3/CC3 analysis. P-values: (*) 0.05≥P>0.01; (**) 0.01≥P>0.001; (***) P≤0.001. Scale bars: (A-D) 375 μm, (E-H) 500 μm, (I-L)150 μm, and (M-BB) 250 μm.

We continued our analysis of these tissue-specific mutants at e14.5. In contrast to the striking midfacial phenotype of both *Kif3a*^*f/f*^;*Wnt1-Cre*, and *Ift88*^*f/f*^;*Wnt1-Cre*, there was no measurable midfacial defect in *Ttc21b*^*f/aln*^;*Wnt1-Cre* embryos (Fig 7A, 7B and 7CC; n = 4). The frontonasal prominence-derived midline of *Ttc21b*^*f/aln*^;*Crect* embryos was dysmorphic, but not significantly wider than that of wild-type embryos (Fig 7C and 7CC; n = 6). The *Ttc21b*^*f/aln*^;*AP2-Cre* frontonasal prominence appeared morphologically normal, yet measured significantly wider than wild-types (Fig 7D and 7CC, n = 4). We next examined the development of the palate in these mutants. The palate of both *Ttc21b*^*f/aln*^;*Wnt1-Cre* and *Ttc21b*^*f/aln*^;*Crect* were cleft, however *Ttc21b*^*f/aln*^;*Wnt1-Cre* palatal shelves appeared to be elevated and patent due to either developmental delay or palatal insufficiency, whereas the palatal shelves in the *Ttc21b*^*f/aln*^;*Crect* were hypoplastic and dysmorphic (Fig 7E–7G). The palate of *Ttc21b*^*f/aln*^;*AP2-Cre* embryos appeared normal (Fig 7H).

Another defining phenotype of *Kif3a*^*f/f*^*;Wnt1-Cre* and *Ift88*^*f/f*^*;Wnt1-Cre* embryos is the presentation of micrognathia/aglossia. Surprisingly, *Ttc21b*^*f/aln*^;*Wnt1-Cre* embryos did not present with either phenotype (S5H Fig). The tongue was clearly present in *Ttc21b*^*f/aln*^;*Crect* embryos, yet there was micrognathia (S5I Fig). The developing mandible of *Ttc21b*^*f/aln*^;*AP2-Cre* embryos resembled that of the *Ttc21b*^*f/aln*^;*Wnt1-Cre* embryos, showing no glossal or lower jaw anomalies (S5J Fig). Unlike *Kif3a* and *Ift88* mutants at this stage, all three *Ttc21b* mutants had a single nasal cartilage (Fig 7I–7L). Thus, *Ttc21b* associated craniofacial phenotypes were less severe than those observed in *Kif3a* and *Ift88* ablations.

We next analyzed each mutant to determine if cell proliferation and cell death were aberrant at e14.5. Within the frontonasal prominence cell proliferation was significantly increased in *Ttc21b*^*f/aln*^;*Wnt1-Cre* embryos, yet unchanged in *Ttc21b*^*f/aln*^;*Crect* or *Ttc21b*^*f/aln*^;*AP2-Cre* embryos (Fig 7M, 7O, 7Q, 7S and 7DD; Table 7). Cell death within the frontonasal prominence was significantly decreased in *Ttc21b*^*f/aln*^;*Wnt1-Cre* and *Ttc21b*^*f/aln*^;*Crect* embryos, yet remained unchanged in *Ttc21b*^*f/aln*^;*AP2-Cre* embryos (Fig 7N, 7P, 7R, 7T and 7EE). Within the e14.5 palate the only significant change observed was increased proliferation in *Ttc21b*^*f/aln*^;*Wnt1-Cre* embryos (Fig 7U–7X and 7FF). None of the three *Ttc21b* mutants had any significant changes in cell death within the developing palate (Fig 7Y–7BB and 7GG).

**Table 7:** Summary of measured values for cell proliferation and cell death in the frontonasal prominence and palatal shelves in e14.5 *Ttc21b* mutants.

Genotype	Stage	Values	PHH3 FNP	CC3 FNP	PHH3 PS	CC3 PS
Control	e14.5	n’s*	24	12	16	15
		Average^+^	44.08	8.94	33.07	8.38
		STD	12.28	2.26	18.64	4.31
		P-value	N/A	N/A	N/A	N/A
*Ttc21b*^*f/aln*^*;Wnt1-Cre*	e14.5	n’s*	17	13	15	13
		Average^+^	58.16	7.01	53.74	12.48
		STD	18.19	2.03	9.81	8.13
		P-value	0.010	0.036	0.0007	0.121
*Ttc21b*^*f/aln*^*; Crect*	e14.5	n’s*	19	14	14	14
		Average^+^	50.34	5.34	39.15	8.48
		STD	13.80	1.56	21.74	3.71
		P-value	0.132	0.0002	0.421	0.944
*Ttc21b*^*f/aln*^*;AP2-Cre*	e14.5	n’s*	12	11	12	10
		Average^+^	49.96	8.66	38.71	5.97
		STD	10.52	2.20	11.68	1.95
		P-value	0.119	0.762	0.334	0.072

frontonasal prominence (FNP), palatal shelves (PS)

* n values indicate number of sections analyzed

^+^ all averages represent the number of PHH3/CC3 positive cells normalized over either FNP or PS area

## Discussion

Ciliopathies are a broad class of diseases that affect various cells and tissues throughout the body. A review by Irigoin and Badano suggested that to fully understand both the biology of cilia and the pathology that arises when they are defective, the organelle must be examined both at different time-points during development and on different cell types [34]. Herein, we addressed this suggestion and evaluated the craniofacial (and neural; see accompanying manuscript by Snedeker et al.) phenotypes that arise when three different ciliary genes were conditionally deleted in various tissues of the craniofacial complex (Table 8). We observed that both tissue and gene identity contributed to the phenotypes produced in the developing face. These findings pose several interesting questions related to the role of cilia and ciliary proteins during development of the face and brain.

**Table 8:** Summary of craniofacial phenotypes, cell proliferation and death trends among conditional ciliary mutants.

Genotype	*Kif3a*^*f/f*^*;Wnt1-Cre*	*Kif3a*^*f/f*^*;Crect*	*Kif3a*^*f/f*^*;AP2-Cre*	*Ift88*^*f/f*^*;Wnt1-Cre*	*Ift88*^*f/f*^*;Crect*	*Ift88*^*f/f*^*;AP2-Cre*	*Ttc21b*^*f/aln*^*;Wnt1-Cre*	*Ttc21b*^*f/aln*^*;Crect*	*Ttc21b*^*f/aln*^*;AP2-Cre*
Midface widening	Yes	Yes	Yes (bimodal) *	Yes	Yes	Yes (bimodal)*	No	No	No
Duplicated nasal septum	Yes	Yes, variable penetrance (33%)	Yes	Yes	NO	Yes	No	No	No
Palatal clefting	Yes	Yes	Yes	Yes	Yes	Yes	Yes, variable penetrance (25%)	Yes	No
Micrognathia	Yes	No	Yes	Yes	No	Yes	No	Yes	No
Tongue defect	Tongue absent	Ectopic growths	Tongue absent	Tongue absent	Ectopic growths	Tongue absent	No	No	No
Cell proliferation- e11.5 FNP	Down	No change	No change	Up	No change	No change	Down	No change	No change
Cell death- e11.5 FNP	No change	Up	No change	No change	Up	No change	Down	Up	No change
Cell proliferation- e11.5 PS	No change	No change	No change	No change	Up	No change	Down	Up	Down
Cell death- e11.5 PS	No change	Up	Up	No change	Down	Down	No change	Up	No change
Cell proliferation- e14.5 FNP	Up	Up	Up	Up	Down	Down	Up	No change	No change
Cell death- e14.5 FNP	No change	Up	No change	Up	Up	No change	Down	Down	No change
Cell proliferation- e14.5 PS	No change	Up	Up	No change	No change	No change	Up	No change	No change
Cell death e14.5 PS	No change	Up	Up	No change	No change	No change	No change	No change	No change

(bimodal)*; percentage of embryos present with a collapsed midline; FNP, frontonasal prominence; PS, palatal shelves

### Severity of phenotype does not linearly correlate to the combination of tissues affected

We set up our experimental design to examine how loss of ciliary function would impact craniofacial development when it occurred in either NCCs (*Wnt1-Cre*), surface ectoderm (*Crect*), or a combination of NCCs, surface ectoderm and neuroectoderm (*AP2-Cre*). We had originally hypothesized that the resulting phenotype from *AP2-Cre* embryos would be the combination of those phenotypes observed with the *Wnt1-Cre* and *Crect* drivers. Interestingly, conditional ablation with *AP2-Cre*, did not consistently have a combinatorial or more severe phenotype than mutants created with *Wnt1-Cre* or *Crect*. There are several explanations for this finding. First, many events in craniofacial development occur as a result of sequential tissue-tissue interactions. For example, signaling centers in the surface ectoderm and neuroectoderm signal to adjacent NCCs during craniofacial development [25, 27, 35]. Thus, loss of cilia in the surface ectoderm or neuroectoderm could affect the recombined tissue itself (autonomous) by altering key signaling centers, and in turn, this could affect the adjacent NCCs (non-autonomous). However, when adjacent tissues lose cilia, as with the *AP2-Cre* driver, tissue-tissue signaling is globally disrupted. This could potentially alleviate some phenotypic presentations as aberrant signals are unable to be received by the adjacent tissue also lacking cilia. Second, the observation that loss of cilia in multiple tissues does not necessary correlate with a more severe phenotype could also be accounted for by the fact that some tissues may utilize cilia to a greater extent than others, thus generating epistatic and hypostatic tissues. Finally, it is possible that the timing of recombination in NCCs and surface ectoderm within the *AP2-Cre* driver occurs slightly later than that in the *Wnt1-Cre* or *Crect*, respectively, thus allowing for some important signaling to occur without incidence. Further molecular analyses must be performed to elucidate how each tissue interprets the loss of functional cilia.

### Conditional knockout of IFT-B genes results in more severe phenotypes than IFT-A genes

Intraflagellar transport (IFT) is a cellular process in which molecular motors transport IFT particles (A and B) and cellular cargo along microtubules. In a ciliary context, IFT is essential for ciliogenesis as it transports tubulin subunits to the tip of growing cilia [36–38]. Within the cilium, IFT-B particles are moved from base-to-tip (anterograde transport) via kinesin-2 motors, whereas IFT-A particles are moved from tip-to-base (retrograde transport) via dynein motors [38–41]. KIF3A is a kinesin-2 motor protein that forms a heterotrimeric complex that is essential for anterograde transport. Furthermore, it is believed to be involved in other cellular processes, including neuronal transport, melanosome movement, and secretory pathway transport [42]. Loss of *Kif3a* results in the complete loss of the axoneme [43]. IFT88 is a member of the IFT-B complex, which also carries cargo in an anterograde direction. Loss of *Ift88* produces truncated cilia in which the axoneme extends just beyond the transition zone [32]. Despite being separate proteins, both KIF3A and IFT88 are essential for ciliogenesis and anterograde IFT. The similarities in their role within the cilium likely account for the similar phenotypes generated when they are conditionally deleted out of various cell types and tissues. Our analyses herein and from our previous work [17]; however, consistently observed that *Kif3a* conditional mutants generated slightly more severe phenotypes (internasal width and degree of nasal septum separation) both phenotypically (Figs 3 and 5) and molecularly [33]. We surmise that the increased severity of phenotypes generated via the loss of *Kif3a* is due to a complete loss of the axoneme for *Kif3a* mutants versus a truncation of the axoneme for *Ift88* mutants.

TTC21B (also known as *Ift139* and *Thm1*) is an IFT-A protein that participates in retrograde transport. The *aln* mutation in *Ttc21b*, which produces a *Ttc21b*-null mutant, generates shorter, wider cilia that have a bulb-like structure at their distal tips [13]. Despite being structurally aberrant, these cilia are not as functionally compromised as those generated via the loss of IFT-B components *Kif3a* and *Ift88*, and thus are likely able to carry out more ciliary function. These findings are consistent with the less severe phenotypes generated in *Ttc21b*^*f/aln*^ mutants (Figs 6 and 7). Currently, there is no ‘characteristic’ phenotype used to diagnose a craniofacial ciliopathy.

However, it is possible that differences in rate of protein degradation between KIF3A, IFT88 and TTC21B following Cre-recombination could also contribute to the variable phenotypes. To definitively test this hypothesis, reliable and robust antibodies for all three proteins would be necessary and protein turn-over assays would have to be performed in each individual tissue. Given that we have documented the loss of cilia after recombination (S2 Fig) [17], we speculate that the phenotypic difference observed are most likely due to the degree to which the cilium is compromised in each mutant. Determining if there is a characteristic phenotype generated depending upon which component of the cilium is compromised (e.g., basal bodies, transition zone or axoneme) could greatly assist in disease diagnosis and therapeutic approaches.

#### Loss of ciliary proteins affects various signaling pathways in distinct ways

Recently, determining the role for the cilium in coordinated signal transduction has dominated research within the field. A plethora of studies have examined how cilia contribute to the signaling of various molecular pathways including Hedgehog, Wnt, PDGF, etc., [3, 5, 41, 44]. For some pathways, receptors are preferentially localized to the ciliary membrane [45–47]. For other pathways, loss of the cilium disrupts the transduction or activity of the pathway itself [13–15, 48–53]. Several of the phenotypes observed in ciliary mutants resemble phenotypes generated when the above mentioned signaling pathways are impaired. A gain of Shh activity [54], loss of Wnt activity [55] or a loss of PDGF [56] activity all produce some degree of midfacial widening, similar to that observed in several of the mutants generated in this study. In light of the established role of cilia in the transduction of multiple signaling pathways as well as the similarities in the phenotypes produced when either the cilia or the signaling pathway is impaired, it is likely that the molecular basis for the phenotypes reported herein are due to a pleiotropic effect on several signaling pathways. Understanding precisely how the cilium transduces these signals, as well as the role of each signal in individual tissues, will be extremely valuable in assessing the basis for ciliopathic phenotypes. Furthermore, determining if specific ciliary proteins have a greater impact on the transduction of some signaling pathways versus others would be of great interest. If this hypothesis were proven true, then targeting the protein’s function, independent of the role in ciliogenesis, would allow for defined manipulation of molecular signaling without impacting the cilium as an organelle. Together, studies such as these could provide new avenues of therapeutic intervention for ciliopathies.

### Are all cilia created equal?

Cilia are frequently referred to as ubiquitous organelles and thought to be highly conserved throughout not only the embryo, but also among various species. Although there is a high degree of conservation, there are specialized cilia within the body including those within the inner ear, the olfactory epithelium, and the retina [57]. What makes these cilia specialized is the cadre of ciliary genes expressed within the cells and tissues they arise from. Thus, for all other cilia within the body, it would be expected that their conserved and ubiquitous nature would be accompanied by conserved and ubiquitous expression of the majority of ciliary genes. Despite this being the dogma of the ciliary field, a significant number of studies report distinct expression patterns for ciliary genes. *Kif3a* is predominantly expressed in brain, although trace amounts of *Kif3a* transcript are detected in various tissues [31]. *Ift88* is most robustly expressed in testis, brain, kidney, lung and pancreas [58, 59]. In contrast, in other vital organs, such as the heart, spleen, and liver, *Ift88* expression is nearly undetectable [58]. In murine embryos, *Ttc21b* is broadly expressed at e6.5 and e7.5 [60]. At e8.5 it maintains a broad expression pattern with more robust levels of expression in the more posterior neural tube and somites. At e9.5–10.5 *Ttc21b* expression can be detected in a number of tissues, but most significantly in limbs, eyes and dorsal neural tube (See accompanying manuscript Snedeker et al.,). Thus, the expression patterns of these three ciliary genes clearly show that their expression is not ubiquitous. Thus, it is likely that cilia in certain regions of the embryo have a unique transcriptome (“ciliome”) that could confer unique function to the cilium, providing an explanation as to why ciliopathies present with a variety of phenotypes.

Our findings within the craniofacial complex, as well as the developing brain (see accompanying manuscript by Snedeker et al.,) suggest that variable phenotypes in ciliopathies are due to unique spatiotemporal expression of ciliary genes or distinct roles for ciliary genes within tissues that contribute to the development of these organ systems. Furthermore, they present an opportunity to study the cilia not as static organelles, but as dynamic signaling hubs that determine how a cell responds to its molecular environment. Our ongoing studies use the mutants generated herein to address these possibilities and aim to determine if modulating expression of certain ciliary proteins can alter the functionality and sensitivity of the cilium to molecular stimuli.

## Materials and methods

### Mouse strains and husbandry

All mouse alleles used in this study have been previously published: *Ttc21b*^*tm1c(KOMP)Wtsi-lacZ*^ (*Ttc21b*^*flox*^) allele [61]; *Kif3a*^*tm2Gsn*^ (*Kif3a*^*flox*^)[62]; *Ift88*^*tm1Bky*^ (*Ift88* ^*flox*^*)*[63]. Timed matings were established and noon on the day of mating plug was designated embryonic day (e) 0.5. This study was carried out in strict accordance with the recommendations in the Guide for the Care and Use of Laboratory Animals of the National Institutes of Health. The protocol was approved by the Institutional Animal Care and Use Committee of the Cincinnati Children’s Hospital Medical Center (protocol number IACUC2013-0113). Animals were housed in ventilated racks with automatic water and feeders providing Purina 5010 autoclavable rodent laboratory chow with a 12 hour light-dark cycles. Certified technical personnel and registered veterinary technicians provide daily observation and handling of lab animals. Signs of dehydration and pain as indicated by hunched and lethargic behavior were monitored to assess animal health. All euthanasia and embryo harvests were performed after isoflurane sedation to minimize animal suffering and discomfort. Animal euthanasia was via cervical dislocation.

### Genotyping

DNA was isolated from tissue samples of embryos. Genotype was determined by PCR using the primer sets listed below. Published protocols were used for all genotyping except for the *Ttc21b*^*aln*^ allele where a custom Taqman assay was employed (Invitrogen; details available upon request). Expected products sizes are denoted in parentheses. *Kif3a*: F-GCTTGTCATCTGGGGAGATT and R-GAACTCCTGGAGGCAGAGG (WT allele- 476 bp, floxed allele- 606 bp); *Ift88* 1-GCCTCCTGTTTCTTGACAACAGTG, 2-GGTCCTAACAAGTAAGCCCAGTGTT, and 3-CTGCACCAGCCATTTCCTCTAAGTCATGTA (WT allele- 350 bp, floxed allele- 370 bp); *Ttc21b*: Aln F: cgctgattaactacta tggtc R: gcgtggtaaaatcggaagac (mutation creates AvaII restriction site). *Ttc21b* flox—F:gcaatgaggtgaccagttttc, R1:ggcgagctca gaccataact R2:agaacaaagcgggacacagt (F+ R2 = wt band, 193bp) and (F+R1 = flox band, 230bp); AP2-Cre F-ATGCCCAAGAAGAAGAGGAAGGT and R-GAAATCACTGCGTTCGAACGCTAGA (product size- 447 bp); Wnt1-Cre F-TAAGAGGCCTATAAGAGGCGG and R- ATCAGTCTCCACTGAAGC (product size- 600 bp); Crect 1- CCT CAC TGA TCC ACA TAT GTC CTT CCG AAA GCT GC, 2- GAT GCT AGA AAG CTG AGG CTG GGC TTA GCT TGC TAG GC; 3- CTA CGC CGC GAA CTT GCT TCT AGA GCG (WT allele- 590 bp, Crect allele- 385 bp)

### Embryo processing

Embryos were harvested at either e11.5 or e14.5, dissected, and imaged. All embryos were fixed in 4% PFA and paraffin embedded. Paraffin sections were cut to 10μm thickness.

### Cell counts

Cell counts were performed using ImageJ software and the Cell Counter feature.

### Imaging equipment

Whole mount images were taken using a Leica M165FC microscope. All other images were taken using a Leica DM5000 B microscope.

### Immunohistochemistry

Immunostaining was performed according to standard protocols. Embryos were fixed in 4% PFA, paraffin embedded and sectioned. Sections were incubated in primary antibody overnight at 4°C. Secondary antibodies with fluorescent tags were then applied at 1:1000 along with Hoechst 33342 (1:2,000; Invitrogen) and incubated at room temperature for 1 hour. Slides were then washed and mounted with mounting media (ProLong Gold, Invitrogen). Antibodies used in this study included: mouse anti-PHH3 (1:500; 05–1336 Millipore), rabbit anti-CC3 (1:500; AF385 R&D Systems) and Peanut agglutinin, FITC conjugate (20 μg/ml; L7381 Sigma).

### Safranin-O staining

Sections were de-paraffinized and rehydrated. Sections were then stained with Weigart’s hematoxylin, rinsed in water and briefly stained with Fast Green (FCF) solution. Sections were rinsed briefly in 1% acetic acid then stained with Safranin-O. Sections were dehydrated and mounted with Permount (Fisher Scientific).

### Statistics

Three embryos for each mutant genotype were collected and sectioned. Staining for cell proliferation and cell death were performed on serial sections. Counts were performed on each section and significance of cell proliferation and cell death were determined using the student’s t-test. Boxplots were generated using BoxPlotR.

## Supporting information

S1 Fig Additional *Crect* recombination analysis. (A, B) Whole-mount e11.5 *Crect;R26R* embryos stained for β-gal. (C-F) Whole-mount e11.5 *Kif3a*^*f/f*^;*Crect;R26R* embryos stained for β-gal. Scale bars: (A,C,E) 575 μm (B, D, F) 500 μm.
(TIF)

S2 Fig Confirmation of Cre driver efficiency. (A-D) Schematic diagram of spatial domain of Cre recombination (blue) for each driver at e11.5. Frontal sections of e11.5 and e14.5 (E, I) wild-type, (F, J) *Kif3a*^*f/f*^*;Wnt1-Cre*, (C,G) *Kif3a*^*f/f*^*;Crect*, and (H, K) *Kif3a*^*f/f*^*;AP2-Cre* embryos immunostained for axonemal marker ARL13B. (E, I) Axonemal extension is detected in both the surface ectoderm and neural crest cells of wild-type animal. (F-H, J-L) Conditional mutants observe a loss of axonemal extension in the neural crest (*Kif3a*^*f/f*^*;Wnt1-Cre*), surface ectoderm (*Kif3a*^*f/f*^*;Crect*), or both tissues (*Kif3a*^*f/f*^*;AP2-Cre*). se: surface ectoderm; oe: oral ectoderm; ncc: neural crest cells. Scale bar = 20 μm.
(TIFF)

S3 Fig Regions analyzed for cell proliferation and cell death. Frontal sections of (A, B) e11.5 and (C, D) e14.5 wild-type embryos. Regions where cell counts were analyzed are outlined with white dotted lines. frontonasal prominence (fnp), meckel’s cartilage (mc), nasal pit (np), nasal septum (ns), palatal shelf (ps), tongue (t).
(TIF)

S4 Fig *AP2-Cre* phenotypic variability. Frontal view of e14.5 (A) wild-type, (B) *Kif3a*^*f/f*^*;AP2-Cre* and (C) *Ift88*^*f/f*^*;AP2-Cre* embryos. Note the hypoteloric midfacial phenotype among the mutants. Saf-O staining on frontal sections through the nasal septum of (D) wild-type, (E) *Kif3a*^*f/f*^*;AP2-Cre* and (F) *Ift88*^*f/f*^*;AP2-Cre* embryos. Scale bar: 375 μm.
(TIF)

S5 Fig Mandibular and tongue development in ciliary mutants. Dorsal views of the developing tongue and mandible at e14.5 in (A) wild-type, (B) *Kif3a*^*f/f*^*;Wnt1-Cre*, (C) *Kif3a*^*f/f*^*;Crect*, (D) *Kif3a*^*f/f*^*;AP2-Cre*, (E) *Ift88*^*f/f*^*;Wnt1-Cre*, (F) *Ift88*^*f/f*^*;Crect*, (G) *Ift88*^*f/f*^*;AP2-Cre*, (H) *Ttc21b*^*f/aln*^*;Wnt1-Cre*, (I) *Ttc21b*^*f/aln*^*;Crect*, (J) *Ttc21b*^*f/aln*^*;AP2-Cre*. Scale bars: 650 μm.
(TIF)

S1 Table Cell counts for PHH3 and CC3 in all mutants at e11.5 and e14.5. Average cell counts, standard deviation and P-values for PHH3 and CC3 staining in epithelial or mesenchymal tissues of FNPs and palatal sections at e11.5 and e14.5. Green boxes indicate significance. N’s refer to the number of sections counted across 3 separate embryos.
(XLSX)
