# Supplementary figures and images for: A tissue-specific role for intraflagellar transport genes during craniofacial development
Source: PLoS One. 2017 Mar 27;12(3):e0174206. doi: 10.1371/journal.pone.0174206 (PMC5367710; doi:10.1371/journal.pone.0174206)

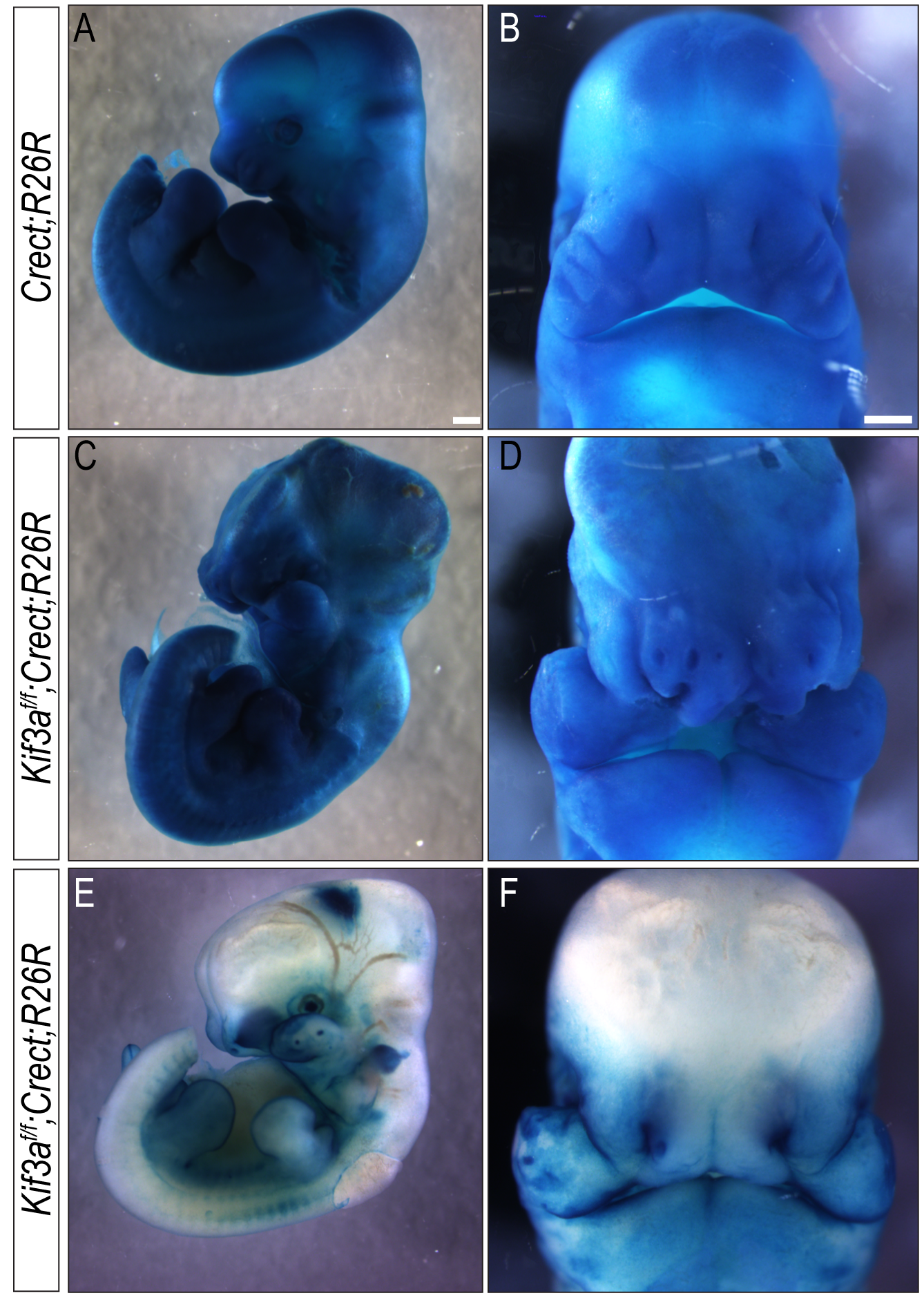

Supplement: S1 Fig — (A, B) Whole-mount e11.5 Crect;R26R embryos stained for β-gal. (C-F) Whole-mount e11.5 Kif3af/f;Crect;R26R embryos stained for β-gal. Scale bars: (A,C,E) 575 μm (B, D, F) 500 μm. (TIF) [file pone.0174206.s001.tif]

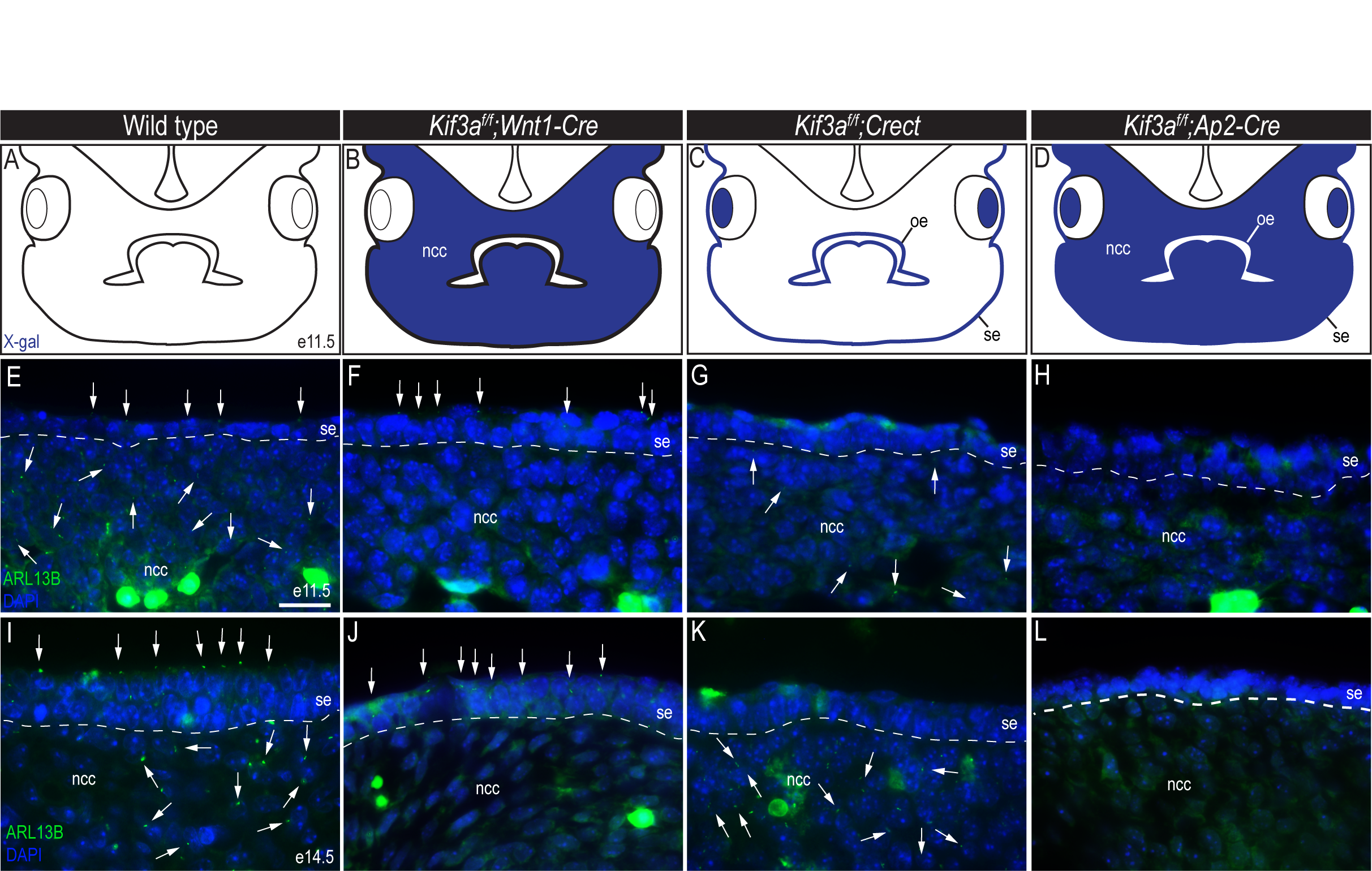

Supplement: S2 Fig — (A-D) Schematic diagram of spatial domain of Cre recombination (blue) for each driver at e11.5. Frontal sections of e11.5 and e14.5 (E, I) wild-type, (F, J) Kif3af/f;Wnt1-Cre, (C,G) Kif3af/f;Crect, and (H, K) Kif3af/f;AP2-Cre embryos immunostained for axonemal marker ARL13B. (E, I) Axonemal extension is detected in both the surface ectoderm and neural crest cells of wild-type animal. (F-H, J-L) Conditional mutants observe a loss of axonemal extension in the neural crest (Kif3af/f;Wnt1-Cre), surface ectoderm (Kif3af/f;Crect), or both tissues (Kif3af/f;AP2-Cre). se: surface ectoderm; oe: oral ectoderm; ncc: neural crest cells. Scale bar = 20 μm. (TIFF) [file pone.0174206.s002.tiff]

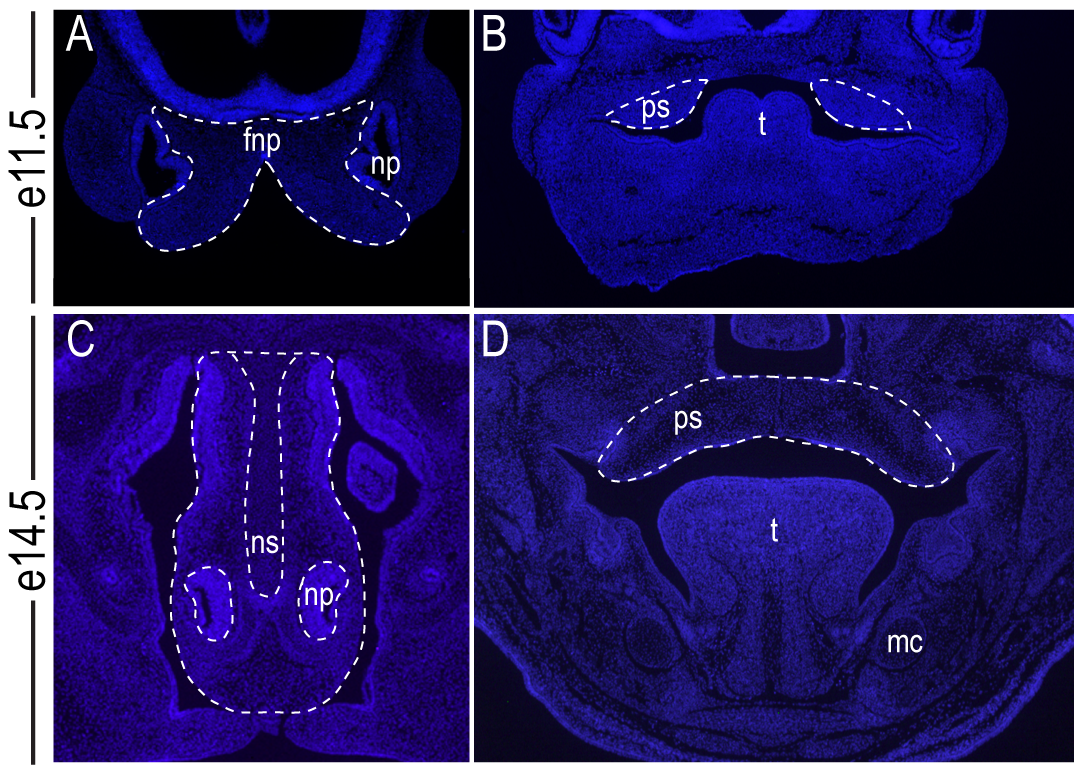

Supplement: S3 Fig — Frontal sections of (A, B) e11.5 and (C, D) e14.5 wild-type embryos. Regions where cell counts were analyzed are outlined with white dotted lines. frontonasal prominence (fnp), meckel’s cartilage (mc), nasal pit (np), nasal septum (ns), palatal shelf (ps), tongue (t). (TIF) [file pone.0174206.s003.tif]

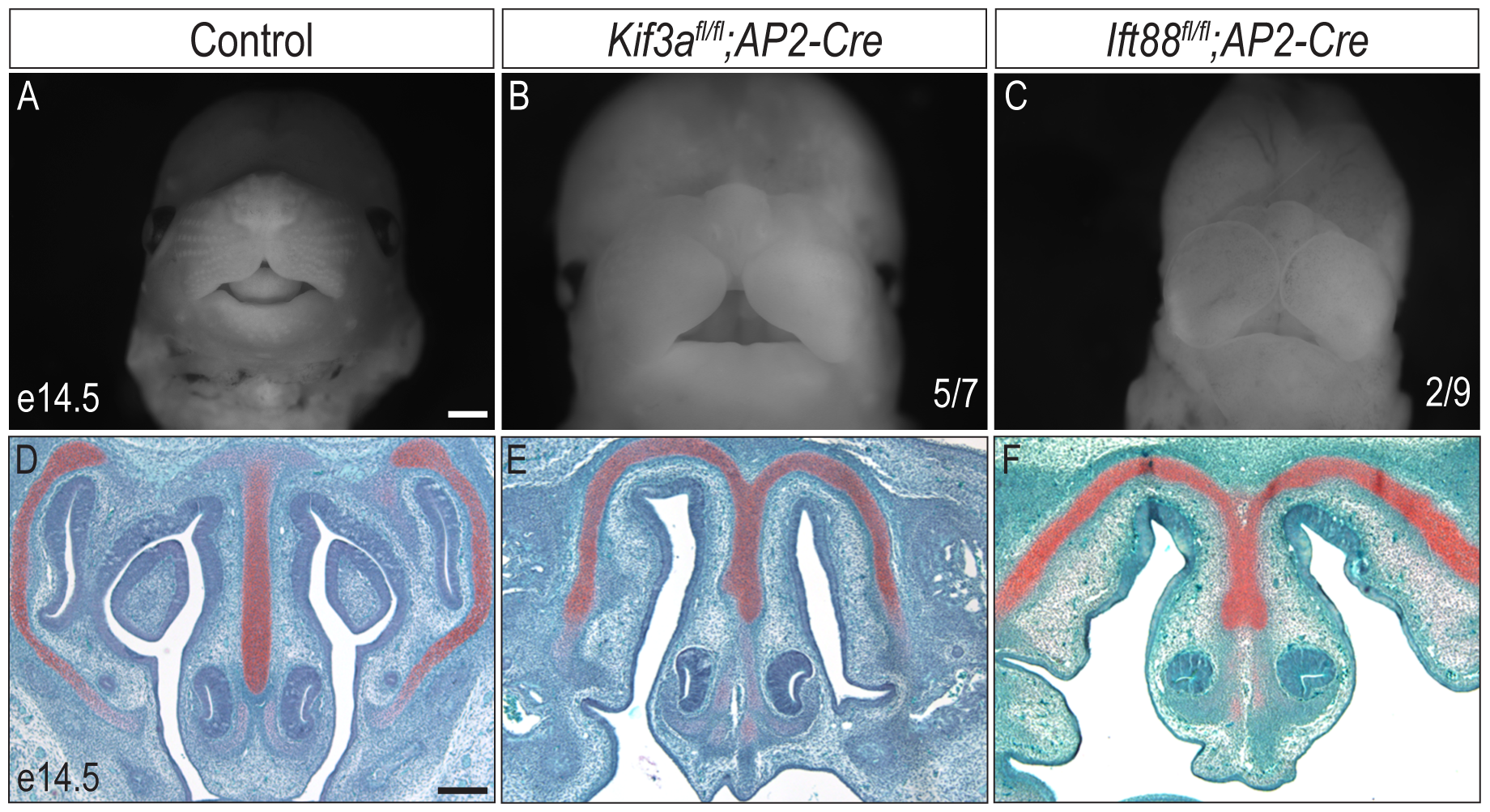

Supplement: S4 Fig — Frontal view of e14.5 (A) wild-type, (B) Kif3af/f;AP2-Cre and (C) Ift88f/f;AP2-Cre embryos. Note the hypoteloric midfacial phenotype among the mutants. Saf-O staining on frontal sections through the nasal septum of (D) wild-type, (E) Kif3af/f;AP2-Cre and (F) Ift88f/f;AP2-Cre embryos. Scale bar: 375 μm. (TIF) [file pone.0174206.s004.tif]

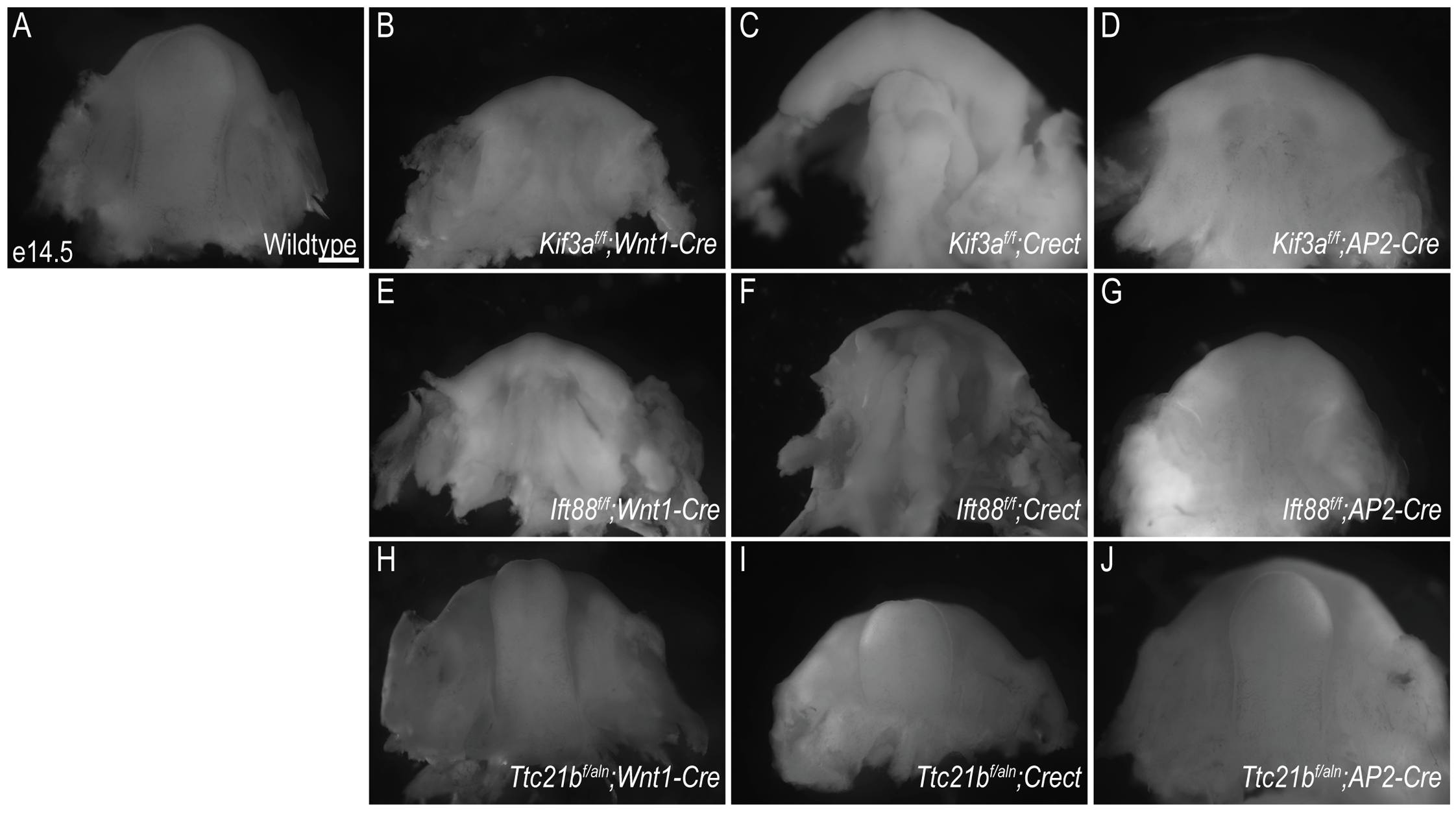

Supplement: S5 Fig — Dorsal views of the developing tongue and mandible at e14.5 in (A) wild-type, (B) Kif3af/f;Wnt1-Cre, (C) Kif3af/f;Crect, (D) Kif3af/f;AP2-Cre, (E) Ift88f/f;Wnt1-Cre, (F) Ift88f/f;Crect, (G) Ift88f/f;AP2-Cre, (H) Ttc21bf/aln;Wnt1-Cre, (I) Ttc21bf/aln;Crect, (J) Ttc21bf/aln;AP2-Cre. Scale bars: 650 μm. (TIF) [file pone.0174206.s005.tif]
